# Structural Control of Metal-Centered Excited States in Cobalt(III) Complexes via Bite Angle and π–π Interactions

**DOI:** 10.1021/jacs.5c09616

**Published:** 2025-07-30

**Authors:** Polina Yaltseva, Tamar Maisuradze, Alessandro Prescimone, Stephan Kupfer, Oliver S. Wenger

**Affiliations:** † Department of Chemistry, 27209 University of Basel, St. Johanns-Ring 19, Basel 4056, Switzerland; ‡ Institute of Physical Chemistry, 9378 Friedrich-Schiller-Universität Jena, Helmholtzweg 4, Jena 07743, Germany

## Abstract

Co^III^ complexes have recently become an important focus in photophysics and photoredox catalysis due to metal-centered excited states with strong oxidizing properties. Optimizing chelate ligand bite angles is a widely used strategy to strengthen metal–ligand interactions in coordination complexes, with the resulting enhanced ligand fields often contributing to extended excited-state lifetimes that are advantageous for photochemical applications. We demonstrate that bite-angle optimization exerts the opposite effect on Co^III^ polypyridines compared to previously studied transition metal complexes, as polypyridine ligands function as π-donors to Co^III^ rather than π-acceptors. Our findings reveal two counterintuitive paradigms: while bite-angle optimization weakens the ligand field in Co^III^ complexes, the resulting lower-energy metal-centered excited states can be accompanied by extended excited-state lifetimes, driven by increased rigidification through intramolecular π–π interactions. These insights, along with additional experiments investigating the possibility of photoreactions from higher excited states, advance the current understanding of the photophysics and photochemistry of first-row transition metal complexes and highlight key distinctions from the more extensively studied photoactive complexes of second- and third-row transition metals.

## Graphical abstract

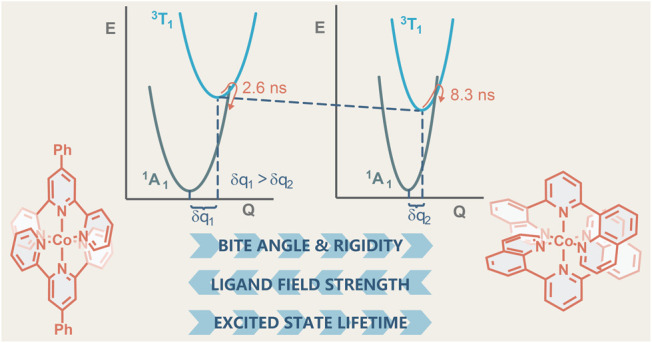

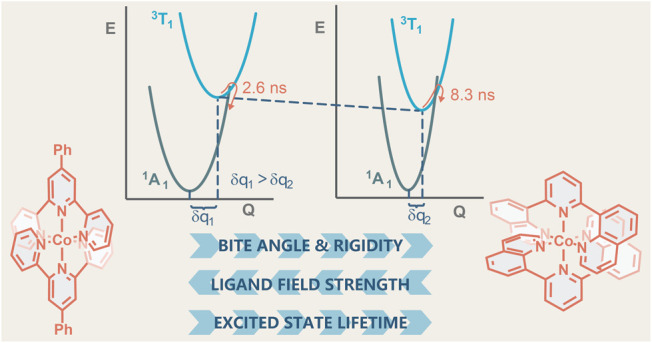

## Introduction

Transition metal complexes with a d^6^ valence electron configuration, such as Ru^II^ (4d^6^), Ir^III^ and Os^II^ (5d^6^), have become essential in applications including solar energy conversion,^1–4^ photocatalysis,^5–10^ light-emitting devices,^11–14^ bioimaging,^15–17^ photodynamic therapy,^18–20^ molecular sensors, and probes.^21–25^ This prominence is attributed to the metal-to-ligand charge transfer (MLCT) excited states of the respective noble metal complexes, which exhibit favorable properties such as high energy storage capacity, redox activity, prolonged excited-state lifetimes, and often excellent luminescence properties. In the pursuit of more cost-effective and earth-abundant alternatives to precious compounds, first-row 3d^6^ transition metals such as Co^III^,^26–31^ Fe^II^,^32–37^ Mn^I^,^38,39^ and Cr^0^,^26,40^ have received substantial attention over the past decade. However, fine-tuning the photophysical properties of 3d metal complexes remains challenging due to the primogenic effect, which leads to a more contracted nature of the 3d metal orbitals compared to the 4d and 5d orbitals, reducing metal–ligand orbital overlap and thus decreasing ligand field strength (10 Dq).^41,42^ This limited orbital overlap leads in consequence to the stabilization of highly distorted metal-centered (MC) states, which further complicates the utilization of charge-transfer (CT) states due to their rapid nonradiative deactivation into the ground state through those MC states.^26,41–45^ Recent studies show that in strong ligand fields, the lowest MC excited states of Co^III^ complexes, although strongly distorted, can become relatively long-lived and photochemically useful.^29,30,46^

A common design strategy for photoactive metal complexes involves using π-acceptor ligands and carefully tuning coordination bite angles to enhance metal–ligand orbital overlap and thereby maximize the ligand field strength.^26,39,42,45,47^ This approach has proven particularly effective in Cr^III^ polypyridine complexes with a d^3^ electron configuration (Figure 1),^48–55^ as it increases the energy of the highly distorted ^4^T_2_ excited state and thereby enables “spin-flip” emission with prolonged lifetimes and enhanced luminescence quantum yields from largely undistorted and energetically lower-lying ^2^E/^2^T_1_ states.

**Figure 1:**
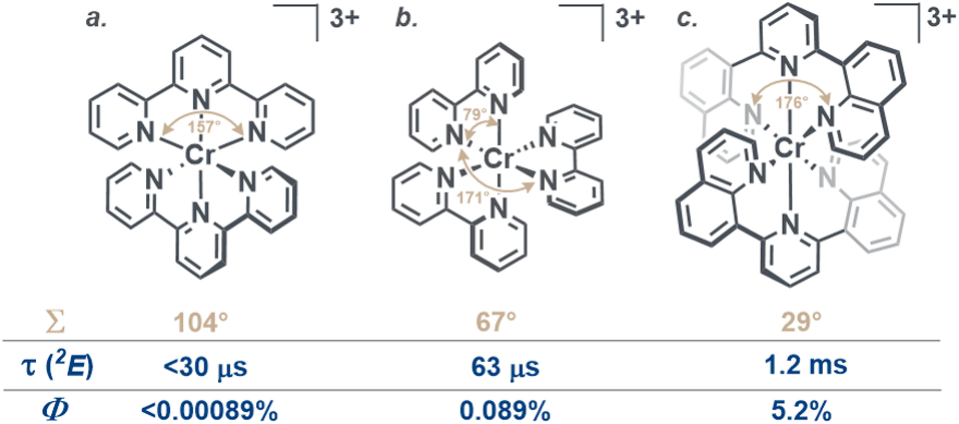
Effect of bite-angle optimization on the lifetimes (τ) of the doublet metal-centered (^2^E) excited state and emission quantum yields (*Φ*) of pseudo-octahedral Cr^III^ polypyridine complexes in solution at room temperature.^56–58^ Selected *cis*- and *trans*-N–Cr–N angles are marked in the molecular drawings to highlight the important geometrical differences between the individual molecular structures. The geometry distortion parameter Σ was calculated as ^12^Σ_i=1_|90 – φ_i_|, where φ_i_ represents *cis*-N–Cr–N angles. The Σ parameter quantifies the deviation from the ideal octahedral geometry.^57,59,60^

Thus, in [Cr­(dqp)_2_]^3+^ (dqp = 2,6-di­(quinolin-8-yl)­pyridine) the increased σ-donation effect associated with its comparatively highly octahedral coordination environment is further combined with the π-acceptor character of the pyridine and quinoline moieties, leading to a large ligand field splitting and consequently an excited-state lifetime (τ­(^2^E)) of 1.2 ms and a photoluminescence quantum yield of 5.2% in solution at room temperature (Figure 1c). The performance of [Cr­(tpy)_2_]^3+^ (tpy = 2,2′:6′,2″-terpyridine) and [Cr­(bpy)_3_]^3+^ (bpy = 2,2′-bipyridine) is exceeded by orders of magnitude (Figure 1a,b).^56–61^ This is primarily due to the stronger Cr^III^–N interactions in [Cr­(dqp)_2_]^3+^, which arise from improved metal–ligand orbital overlap enabled by a coordination geometry that more closely approximates an ideal octahedron compared to [Cr­(tpy)_2_]^3+^ and [Cr­(bpy)_3_]^3+^.^58^ The deviation from the ideal geometry can be quantified by the octahedral distortion parameter Σ, which is defined as the sum of deviations from 90° for all 12 *cis*-N–Cr–N angles (^12^Σ_i=1_|90 – φ_i_|).^62^ The Σ parameter decreases from 104° to 67° and then to 29° across the series of complexes shown in Figure 1, resulting in significant enhancements in τ­(^2^E) and Φ as described above.

Beyond the d^3^ complexes of Cr^III^ and Mn^IV^,^63–65^ strong ligand fields generated by π-acceptor ligands and chelates with optimized bite angles have been most effective in complexes of d^6^ metals. In Cr^0^ and Mn^I^ complexes,^38,40,66–69^ the combined σ-donating and π-accepting electronic properties of isocyanide chelate ligands, paired with nearly ideal octahedral geometries, make it possible to achieve emissive ^3^MLCT excited states with lifetimes in the nanosecond range at room temperature in solution.^38,40,66–69^

Among photoactive 3d^6^ systems, Fe^II^ complexes have received by far the most attention, and efforts to modify their photophysical properties have also focused on creating strong ligand fields and idealized coordination geometries.^32,36,70–87^ In pursuit to extend the ^3^MLCT excited state lifetimes of Fe^II^ complexes, several research groups have developed ligands based on N-heterocyclic carbene (NHC) and mesoionic carbene (MIC) moieties, which can act as strong σ-donors and π-acceptors, increasing the ligand field strength.^43,47,75,79,81,82,85,88^ Ultimately, this molecular design extended the ^3^MLCT lifetime up to ∼500 ps in the 6-fold MIC coordination environment of [Fe­(btz)_3_]^2+^ (btz = 3,3′-dimethyl-1,1′-bis­(p-tolyl)-4,4′-bis­(1,2,3-triazol-5-ylidene)), about 5000 times longer than in the benchmark [Fe­(bpy)_3_]^2+^ complex.^82^

The higher oxidation state of Co^III^ relative to Fe^II^ inherently generates a stronger ligand field, providing a priori an advantageous electronic environment.^89,90^ Recent work demonstrated how to control ^3^MC excited state deactivation in Co^III^ polypyridine complexes, leading to promising application potential of these states in photoredox catalysis.^29,30,46,91^ In [Co­(4,4′-R_2_bpy)_3_]^3+^ complexes the relationship between ligand field strength and the lifetimes of ^3^MC excited states was rationalized in terms of Marcus inverted region behavior,^92^ analogous to the common behavior of ^3^CT excited states in Ru^II^ or Os^II^ complexes.^93^ In this regime, an increase in the excited state energies leads to a decrease in the (nonradiative) deactivation rate of the excited state. This finding is essential for designing Co^III^ systems possessing long-lived ^3^MC excited states with high energy capacity, which are crucial for photocatalytic applications. Furthermore, this behavior contrasts with that commonly seen for isoelectronic Fe^II^ complexes.^91^ Previous observations on [Co­(PhB­(MeIm)_3_)_2_]^+^ (PhB­(MeIm)_3_^–^ = tris­(3-methylimidazolin-2-ylidene)­phenyl borate) and [Co­(CN)_6_]^3–^ complexes align well with the introduced concept of Marcus inverted behavior. In these complexes, the very strong ligand field splitting due to the 6-fold NHC or cyanido environments caused the ^3^MC states to become emissive, exhibiting lifetimes of 1 μs and 2.6 ns, respectively, owing to the sufficiently large energy gap between these states and the ground state (∼1.7–1.8 eV).^94–98^ However, the impact of coordination bite angles on the photophysical properties of Co^III^ polypyridine complexes remains unexplored.

In the present work, we focus on investigating how the properties of ^3^MC excited states in Co^III^ complexes are influenced by modification of the ligand bite angles, expansion of the aromatic π-system of the ligand framework, and cooperative rigidity due to π–π and steric interactions between individual ligands coordinated to the same metal center. While it is well established for Cr^III^ that σ-donation and π-backbonding play key roles in the ligand design based on polypyridine scaffolds (Figure 2a,b), comparatively little is known about those effects in Co^III^ complexes.^48,57^

**Figure 2:**
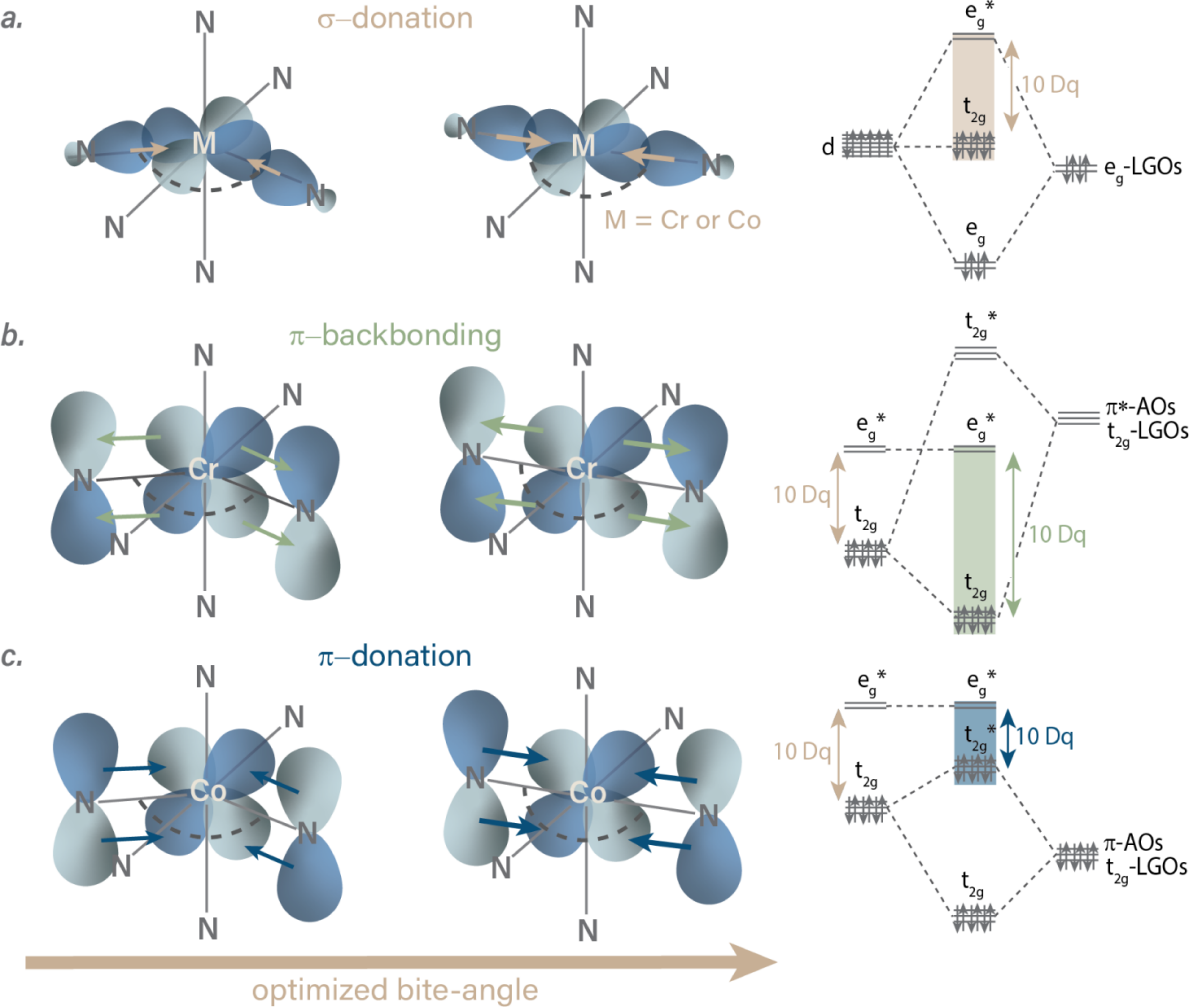
Anticipated main electronic effects upon bite-angle optimization shown in a simplified orbital scheme: a. σ-donation in Cr^III^ and Co^III^ polypyridine complexes, b. π-backbonding in Cr^III^ polypyridine complexes, c. π-donation in Co^III^ polypyridine complexes. In a–c molecular orbital diagrams show relevant bonding interactions of metal 3d orbitals and (symmetry-adapted) ligand group orbitals (LGOs).^43^ The magnitude of the illustrated electronic effects increases as the coordination geometry approaches a more ideal octahedral arrangement.

We hypothesize that improving the metal–ligand orbital overlap via enhancing the ligand bite angles for an octahedral geometry can enhance σ-donation, similar to the case for Cr^III^ complexes (Figures 1 and 2a). However, literature reports indicate that polypyridine ligands behave as π-donors rather than π-acceptors toward Co^III^, which would be expected to stabilize MC states (Figure 2c) and could entail shorter excited-state lifetimes.^99,100^ To experimentally test this hypothesis, we compare the photophysical properties of compounds of known structure—[Co­(phtpy)_2_]^3+^ (phtpy = 4′-phenyl-2,2′:6′,2″-terpyridine) and [Co­(dqp)_2_]^3+^ (Figure 3a),^101–103^ where the differences between coordination geometries are similarly pronounced to those observed in the Cr^III^ complexes shown in Figure 1a,c. Our hypothesis is confirmed to the extent that π-donation, rather than π-acceptor behavior, predominates in the Co^III^ complexes; however, the resulting stabilization of the ^3^MC excited state does not lead to the anticipated shortening of its lifetime but rather to an unexpected extension. This finding suggests enhanced cooperative rigidity arising from the extended π-conjugated framework of the dqp ligand, which enables intramolecular π–π interactions that rigidify the overall coordination complex and reduce the efficiency of nonradiative ^3^MC relaxation.^104–106^ The observed simultaneous lowering of excited-state energy and prolongation of its lifetime contradicts the typical photophysical behavior seen in most metal complexes and organic compounds but can be attributed to a rationalizable interplay between ligand field strength, excited-state distortions, and force constants.

**Figure 3:**
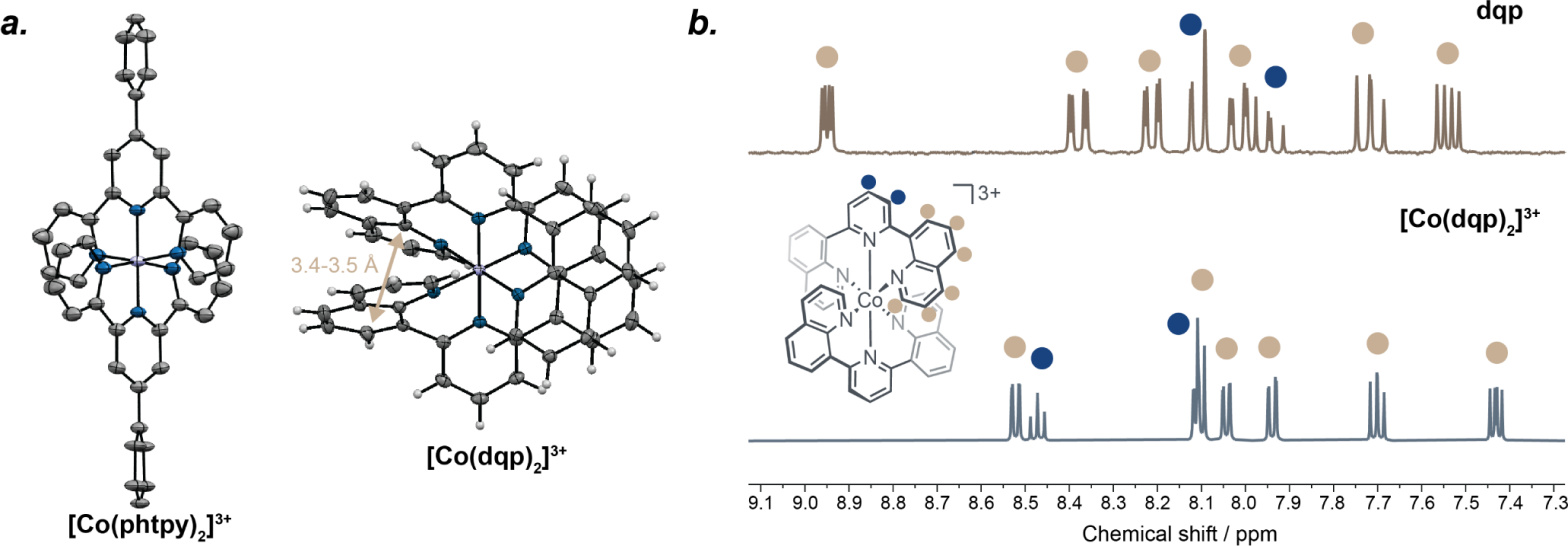
(a) X-ray crystal structures of [Co­(phtpy)_2_]^3+^,^102^ and [Co­(dqp)_2_]^3+^ (Supporting Information); counterions and solvent molecules are omitted for clarity, and thermal ellipsoids are shown at 50% probability; π–π interactions are indicated with distance (Å) between the quinoline units. (b) ^1^H NMR spectra of the free dqp ligand (top) and [Co­(dqp)_2_]^3+^ in CD_3_CN (bottom), signals corresponding to the protons of the quinoline moieties, involved in π–π interactions, are marked in brown, while the protons of the pyridine unit are marked in blue.

## Results and Discussion

### Two Sister Complexes with Different Coordination Geometries

The 4′-phenyl-2,2′:6′,2″-terpyridine ligand (phtpy) is synthetically more accessible than unsubstituted tpy, so we used phtpy instead of tpy for our study but expect the primary coordination sphere to be largely unaffected by the additional phenyl ring in the backbone. The phtpy ligand and the compound [Co­(phtpy)_2_]­(PF_6_)_3_ were synthesized following procedures adapted from the literature (Supporting Information).^102,107^ [Co­(dqp)_2_]­(PF_6_)_3_ was synthesized in a manner similar to [Co­(phtpy)_2_]­(PF_6_)_3_.^101,103^ In the first step, the dqp ligand (2.05 equiv) reacted with CoCl_2_·6H_2_O (1.0 equiv) in CH_3_CN, followed by Co^II/III^ oxidation with Br_2_, and finally, the anion was exchanged with KPF_6_ in aqueous solution. After purification, the complex was characterized by standard analytical techniques—NMR spectroscopy, high-resolution mass spectrometry, and combustion analysis (Supporting Information).

Orange crystals of [Co­(dqp)_2_]­(PF_6_)_3_·2CH_3_CN, suitable for X-ray diffraction (XRD) measurements, were obtained via vapor diffusion of Et_2_O into a saturated CH_3_CN solution. Analysis of the crystal data revealed the Co–N bond lengths to be in the range of 1.952(2)–1.972(2) Å, in line with the values for previously reported tridentate polypyridine complexes.^107^ Compared to [Co­(phtpy)_2_]^3+^, upon extension of the aromatic system in [Co­(dqp)_2_]^3+^, bite angles increase from 164.7(3)° to 179.42(10)°–179.21(10)°, approaching the ideal octahedral 180°, similar to what has been reported previously for [Ru­(dqp)_2_]^2+^,^108–110^ and for Cr^III^ complexes with bite-angle optimized chelate ligands.^48,49,57,110,111^ The analysis of the octahedral distortion parameter Σ reveals a highly octahedral coordination environment with a Σ = 13° in [Co­(dqp)_2_]^3+^ as opposed to a Σ = 65° in [Co­(phtpy)_2_]^3+^. Interestingly, the Σ value in [Co­(dqp)_2_]^3+^ is lower than in [Cr­(dqp)_2_]^3+^ (Σ = 29°),^57^ presumably due to a smaller ionic radius of Co^III^ compared to Cr^III^.^62^

These structural properties as obtained in the solid state are in good agreement with the fully relaxed, calculated singlet ground-state structure of [Co­(dqp)_2_]^3+^ in acetonitrile. Density functional theory (DFT) predicts Co–N bond lengths ranging from 1.961 to 1.978 Å, while a bite angle of 178.7° is obtained. All calculated equilibrium structures (as well as high-resolution images) are available via the free online repository Zenodo.^112^ In the crystal structure of [Co­(dqp)_2_]­(PF_6_)_3_ π-stacking effects of the adjacent ligand “side-arms,” namely the quinoline units, are evident (Figure 3a), which could contribute to a favorable rigidity-enhancing effect.^105^ π–π interactions are indicated by roughly 3.4–3.5 Å distances between the relevant quinoline centroids (Figure 3a),^113^ matching the values reported for [Cr­(dqp)_2_]^3+^.^57^ Notably, the structure exhibits helical twisting, compatible with P- and M-chirality according to Cahn–Ingold–Prelog nomenclature,^114^ and pairs of PP- and MM-enantiomers are present in the crystal unit cell, reminiscent of what has been reported for [Cr­(dqp)_2_]^3+^ and related Cr^III^ complexes.^49,57,115^

Evidence for the intramolecular π-stacking effects between individual dqp ligands is further provided by comparison of the ^1^H NMR spectra of [Co­(dqp)_2_]^3+^ and the free dqp ligand (Figure 3b). Upon coordination, the quinoline protons (marked in brown in Figure 3b) of the ligand experience a drastic upfield shift, while the pyridine protons (marked in blue in Figure 3b) undergo a downfield shift. Typically, without any structural constraints, a downfield shift is anticipated for the ligand protons due to an electron density displacement from the ligand to the metal upon coordination. However, the observed upfield shift of the quinoline protons indicates that those units are likely located in close proximity to one another. These data are also consistent with π–π interactions observed in the ^1^H NMR spectrum of [Ru­(dqp)_2_]^2+^.^108^

Cyclic voltammetry of [Co­(dqp)_2_]^3+^ in dry and deaerated CH_3_CN with 0.1 M (*n*-Bu_4_N)­PF_6_ revealed two reversible reduction events (Figure S1). The first wave at +0.33 V vs SCE (*E*_red_, see below) can be assigned to the Co^II/III^ redox pair and the second wave at −0.99 V vs SCE can be attributed to a ligand-based reduction process. The assignment of the metal-centered reduction is supported by the quantum chemical simulations, which reveal the $\sigma_{x^{2}{-y}^{2}}^{*}$ character (or $d_{x^{2}{-y}^{2}}$ in an atomic orbital picture) of the lowest unoccupied molecular orbital (LUMO) of the [Co­(dqp)_2_]^3+^ complex, while the LUMO-1—associated with the second reduction—is of π*_dqp_ nature (Figure S23). When comparing the electrochemical behavior of [Co­(dqp)_2_]^3+^ to that of other Co^III^ polypyridine complexes, the metal reduction event was observed at very similar potentials of +0.32 V vs SCE and +0.26 V vs SCE in [Co­(4,4′-Br_2_bpy)_3_]^3+^ and [Co­(phtpy)_2_]^3+^, respectively.^46,102,116^

### Bite-Angle Optimization Weakens the Ligand Field

In the UV–vis absorption spectra of [Co­(phtpy)_2_]^3+^ and [Co­(dqp)_2_]^3+^, the strongest bands are observed below 400 nm (Figure 4a,b). In the case of [Co­(phtpy)_2_]^3+^ and in agreement with the experimental observations our TD-DFT simulations associate the main absorption feature at 340 nm with a strongly dipole-allowed intraligand charge transfer (ILCT, ph → tpy) into S_4_ (at 3.27 eV, 378 nm, Figure 4c and Table S4). Similarly, [Co­(dqp)_2_]^3+^ shows an absorption band with mixed LC/LMCT character (LC = π–π* ligand-centered, LMCT = ligand-to-metal charge transfer) at slightly lower energy, appearing at 360 nm (ε = 26,000 M^–1^ cm^–1^), while the main electronic transition, predicted at 369 nm (into S_12_, 3.36 eV, Figure 4d and Table S8), is related to a local excitation of the π-system of the coordinated dqp ligands with only very minor LMCT character. The bathochromic shift of the absorption band at 360 nm in [Co­(dqp)_2_]^3+^ compared to that at 340 nm in [Co­(phtpy)_2_]^3+^ is likely the result of the increased aromatic system on the dqp ligand.

**Figure 4:**
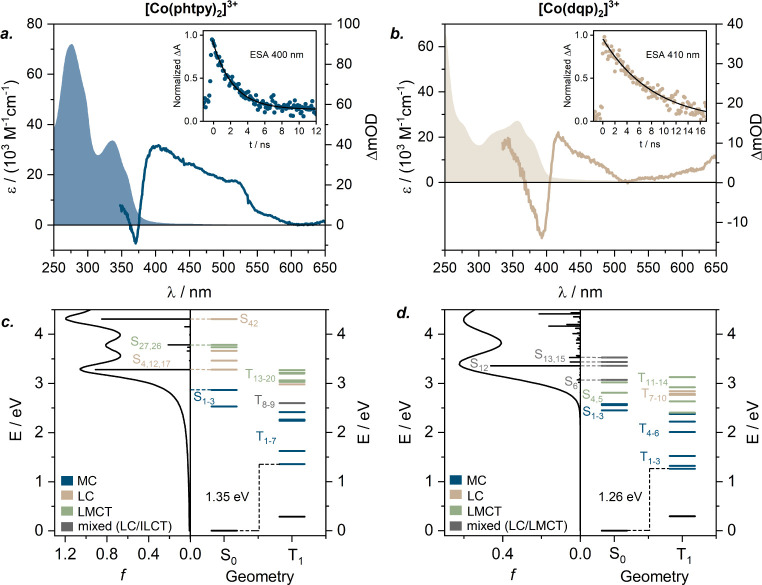
Top row: Experimental UV–vis absorption spectra in CH_3_CN (filled); experimental UV–vis transient absorption spectra in deaerated CH_3_CN at room temperature following excitation at 355 nm with picosecond pulses (∼6 mJ per pulse) time-integrated over 20 ns (solid traces); kinetic transient absorption traces at the indicated wavelengths, and their monoexponential fitting results (insets). The individual data sets correspond to a. [Co­(phtpy)_2_]^3+^ and b. [Co­(dqp)_2_]^3+^; TD-DFT calculated state energies (S_0_ – singlet ground state geomerty, T_1_ – first excited triplet state geometry, corresponding to the ^3^T_1_ excited state) with their character assignment (see the insets for the color coding), and simulated ground-state absorption spectra for the respective complexes: c. [Co­(phtpy)_2_]^3+^; d. [Co­(dqp)_2_]^3+^.

Additionally, weak absorption bands observed in both spectra in the visible region beyond 400 nm, with a molar absorptivity below 500 M^–1^ cm^–1^, can be attributed to the spin-forbidden MC transitions. Gaussian deconvolution analysis of those weak bands in both [Co­(phtpy)_2_]^3+^ and [Co­(dqp)_2_]^3+^ (Figure 5), allowed to determine the ^1^MC (^1^T_1_) and ^3^MC (^3^T_1_) transition energies, required for the ligand field parameter analysis. The mixed ^1^MC states S_1_–S_3_ in [Co­(phtpy)_2_]^3+^ are predicted by the calculations at 490–432 nm (2.53–2.87 eV) with slight LMCT contributions, similarly in [Co­(dqp)_2_]^3+^ the ^1^MC states appear at 506–480 nm (S_1_–S_3_, 2.45–2.58 eV), while ^1^LMCT states are predicted at 442 and 410 nm (S_4_ and S_5_, 2.81 and 3.02 eV), see Tables S4 and S8 for details.

**Figure 5:**
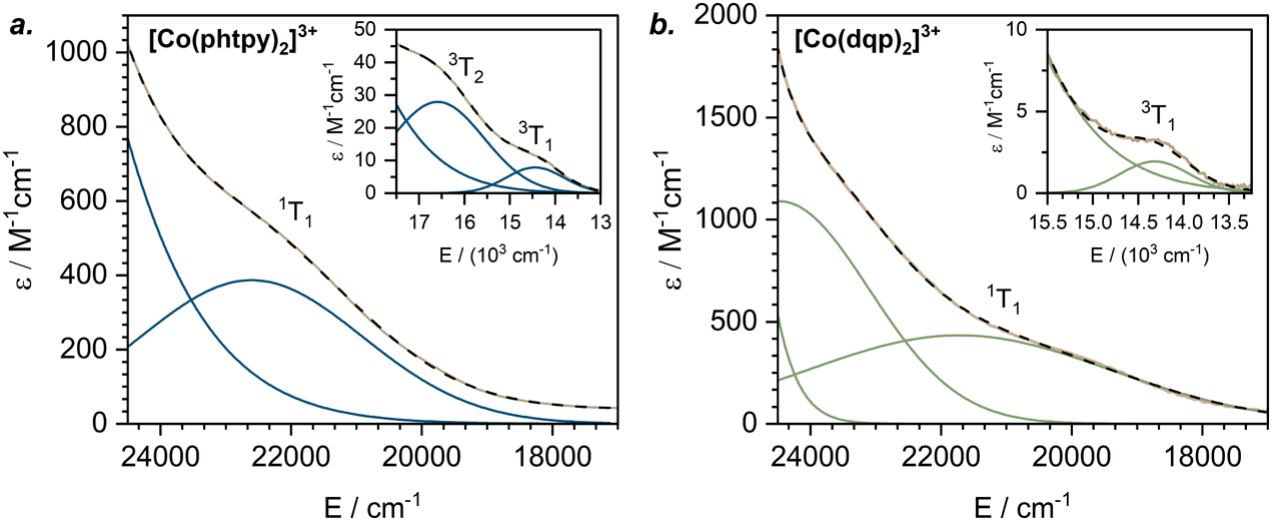
Low-energy parts of the experimental UV–vis absorption spectra of a. [Co­(phtpy)_2_]^3+^ and b. [Co­(dqp)_2_]^3+^ in CH_3_CN (brown) with Gaussian deconvolution and assignment of the ^1^MC (^1^T_1_) and ^3^MC (^3^T_1_ and ^3^T_2_) electronic transitions. The cumulative fitted curve is shown with dashed black lines. MC transition energies: a. Δ*E* (^1^T_1_–^1^A_1_) = 22,600 cm^–1^, Δ*E* (^3^T_2_–^1^A_1_) = 16,600 cm^–1^, Δ*E* (^3^T_1_–^1^A_1_) = 14,500 cm^–1^; b. Δ*E* (^1^T_1_–^1^A_1_) = 21,700 cm^–1^, Δ*E* (^3^T_1_–^1^A_1_) = 14,300 cm^–1^.

We could not confidently determine the energies of all of the MC transitions required for a precise estimation of the 10 Dq and Racah parameters in [Co­(dqp)_2_]^3+^ and [Co­(phtpy)_2_]^3+^ due to the tailing of intense CT transitions in the same spectral range. However, the observed electronic transitions from the singlet ground state (^1^A_1_) to the ^1^T_1_ and ^3^T_1_ excited states allowed an estimation of 10 Dq and the Racah parameter C for both Co^III^ complexes (see Figure 5 and the footnote of Table 1 for details).^117^ The relevant parameters for the [Co­(bpy)_3_]^3+^ complex have been reported previously,^99^ enabling a comparison of 10 Dq values across the series [Co­(phtpy)_2_]^3+^, [Co­(bpy)_3_]^3+^, and [Co­(dqp)_2_]^3+^. Within this series, the symmetry around the Co^III^ center becomes progressively more octahedral while the Σ parameter decreases (Table 1), mirroring the trend observed for the Cr^III^ complexes shown in Figure 1. For the 10 Dq values, however, opposite trends are observed for Co^III^ and Cr^III^. Whereas 10 Dq increases for Cr^III^ across the ligand series tpy < bpy < dqp (as discussed in the Introduction), it decreases for Co^III^ along the series phtpy > bpy > dqp (Table 1). This fundamentally different behavior of Cr^III^ and Co^III^ complexes can be rationalized by taking into account the effective nuclear charges. Due to its smaller ionic radius, Co^III^ has a higher effective nuclear charge, resulting in lower energies of its t_2g_ orbitals and a closer energy alignment with the ligand π-orbitals than in the case of Cr^III^.^99^ Consequently, while the tpy/phtpy, bpy, and dqp ligands act as π-acceptors to Cr^III^, they behave as π-donors toward Co^III^.^99^ Whereas π-acceptors enhance the ligand field strength, π-donors lead to weaker ligand fields (Figure 2b,c), consistent with the trends observed in Table 1.

**Table 1:** Ligand Field Parameters, Racah Parameters, and Octahedral Distortion Parameters in Co^III^ and Cr^III^ Complexes along with the Lifetimes of Their Lowest Electronically Excited States^29,49,56,57,61,99^ ^a^

Complex	10 Dq^†^/cm^–1^	C^†^/cm^–1^	τ(^3^T_1_)/ns	Σ/°	Complex	10 Dq/cm^–1^	τ(^2^E)/μs	Σ/°
[Co(phtpy)_2_]^3+^	26,650	4050	2.6^b^	65	[Cr(tpy)_2_]^3+^	20,390 ^49^	30^c^ ^56^	104
[Co(bpy)_3_]^3+^	25,900 ^99^	3730 ^99^	5.0^b^ ^29^	47	[Cr(bpy)_3_]^3+^	23,300 ^49^	63^c^ ^56^	67
[Co(dqp)_2_]^3+^	25,400	3700	8.3^b^	13	[Cr(dqp)_2_]^3+^	24,900 ^57^	1200^b^ ^57^	29

a 10 Dq and Racah parameter *C* values for [Co­(phtpy)_2_]^3+^ and [Co­(dqp)_2_]^3+^ were approximated using the Tanabe–Sugano formalism:^117^ Δ*E*(^3^T_1_–^1^A_1_) = 10 Dq – 3*C*, Δ*E* (^1^T_1_–^1^A_1_) = 10 Dq – *C* (experimental values were extracted from the data in Figure 5).

b Acetonitrile solution, room temperature.

c Aqueous solution, room temperature.

This is in line with recent work demonstrating that bidentate and tridentate polypyridine ligands in Co^III^ complexes primarily act as π-donors.^99^ This behavior contrasts the π-acceptor character of polypyridines in second- and third-row transition metal complexes,^118^ and supports the previously observed trend that [Co­(tpy)_2_]^3+^, with its more distorted bite angles, exhibits a stronger ligand field splitting than [Co­(bpy)_3_]^3+^ (Table 1).^99^ McCusker and colleagues^99^ proposed that this difference between tpy and bpy complexes arises due to a weaker π-donation by the tpy ligand owing to less favorable metal–ligand orbital overlap (Figure 2b), leading to a weaker destabilization of the t_2g_^*^ orbitals and a (slightly) stronger ligand field in the tpy complex compared to the bpy complex (Figure 2c).^99^ Following that logic in our case, the π-donation is strongest with the bite-angle optimized dqp ligand, leading to the weakest ligand field. The calculated triplet energy of [Co­(dqp)_2_]^3+^ lies at 1.26 eV (see T_1_ in Figure 4d), reflecting an approximately 0.1 eV stabilization of the lowest ^3^T_1_ state compared to 1.35 eV in [Co­(phtpy)_2_]^3+^ (see T_1_ Figure 4c). The calculated decrease in the ^3^T_1_ energy between these two bis­(tridentate) coordination environments thus aligns with the experimentally observed decrease of 10 Dq, at least in terms of the overall trend.

### Prolonged Excited-State Lifetimes Despite Lower Energies

The excited-state dynamics of the Co^III^ complexes were investigated via picosecond UV–vis transient absorption spectroscopy in deaerated acetonitrile at room temperature and in combination with computational modeling. Following the excitation of [Co­(phtpy)_2_]^3+^ at 355 nm, multiple excited-state absorption (ESA) bands could be observed throughout the visible region (Figure 4a), decaying with a lifetime of 2.6 ns. In this case, TD-DFT simulations enabled the assignment of these ESA spectral signatures to electronic transitions from the lowest ^3^MC state to higher-lying excited states. In particular, the ESA in [Co­(phtpy)_2_]^3+^ was associated with a low-lying ^3^LMCT transition (into T_9_ at 647 nm) as well as with ^3^ILCT excitation into T_36_ at 379 nm (Figure S20 and Table S6).

Shifting our focus to the complex with an optimized bite angle [Co­(dqp)_2_]^3+^, its TA spectrum is also compatible with the lowest excited state of ^3^MC character (Figure S22), with a lifetime of 8.3 ns. ESA bands of the ^3^MC state are observed at 410, 465, and 650 nm (Figure 4b), along with a ground-state bleach (GSB) at 390 nm, corresponding to the LC/LMCT band in the ground-state absorption spectrum. The computational analysis assigns these ESA bands mostly to an LMCT absorption at 627 nm (T_11_), several highly mixed and weakly allowed transitions (e.g., T_31_, T_33_ and T_35_ between 432 and 402 nm) as well as with a strongly allowed LC transition (T_54_, 364 nm; Figure S22 and Table S10).

Across the series of Co^III^ complexes reported in Table 1, the lifetime of the lowest ^3^T_1_ excited state increases as the ligand field strength decreases (i.e., as 10 Dq decreases) and the energy of the ^3^T_1_ state becomes lower. Specifically, the ^3^T_1_ lifetime increases from 2.6 ns in [Co­(phtpy)_2_]^3+^ to 5.0 ns in [Co­(bpy)_3_]^3+^ ^29^ and to 8.3 ns in [Co­(dqp)_2_]^3+^ (Table 1, Figure 5a,b). This contrasts the Marcus inverted region behavior recently unraveled for polypyridine complexes of Co^III^, where an increase of the ^3^MC energy led to an increase of the ^3^MC lifetime.^29,30^ It seems plausible that the discrepant behavior observed here for [Co­(phtpy)_2_]^3+^ and [Co­(dqp)_2_]^3+^ could be due to different extents of structural rearrangements in the ^3^MC excited state relative to the electronic ground state. According to its X-ray crystal structure and the DFT simulations in a solvent environment, [Co­(phtpy)_2_]^3+^ (Figure 3a) has a strong geometric distortion in the electronic ground state (Σ = 65°, Figure 3a and Table 1) due to the strained bite angle (164(10)°) and axial compression, with the two axial Co–N bonds of 1.862(7) Å being considerably shorter than the four equatorial Co–N bonds (1.952(5) Å).^102^

By contrast, the ground state of [Co­(dqp)_2_]^3+^ shows minor deviations from the ideal octahedral geometry (Σ = 13°, Figure 3a and Table 1) and reveals significant π-interactions between individual dqp ligands coordinated to the same metal (Figure S23, MOs 165–178), which could impart structural rigidity and greater force constants associated with molecular distortions both in the ground state and in the ^3^MC excited state.^105^ This in turn could lead to more nested ^3^T_1_/ground state potentials (Figure 6b), making the ^3^T_1_ lifetime more than a factor of 2 longer in [Co­(dqp)_2_]^3+^ than in [Co­(phtpy)_2_]^3+^, despite a decrease in triplet energy of about 0.1 eV (see above). The ca. 40% increase in the ^3^T_1_ lifetime from [Co­(phtpy)_2_]^3+^ to [Co­(bpy)_3_]^3+^ is comparatively modest and more difficult to rationalize within a simple qualitative framework. This is especially true given the difference in coordination environments (bis­(tridentate) versus tris­(bidentate)), which complicates direct comparisons. Therefore, it seems more appropriate to focus the following discussion on the tridentate systems, specifically comparing phtpy/tpy-based and dqp-based complexes.

**Figure 6:**
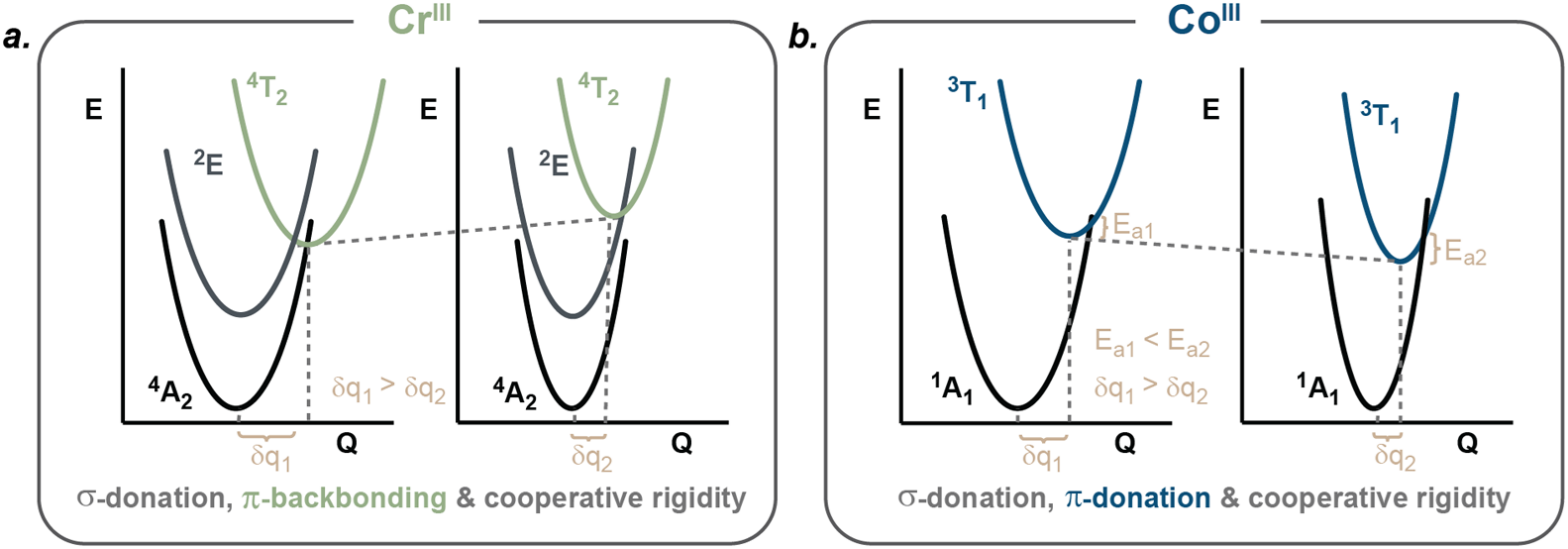
Main electronic and cooperative rigidity effects in Cr^III^ and Co^III^ complexes upon bite-angle optimization and extension of the π-conjugation. Anticipated changes in the energies, nuclear coordinates, and force constants of the electronic ground and relevant excited states upon optimization of bite angles, π-conjugation, and cooperative rigidity in simplified illustrations with harmonic potential well diagrams. Q = nuclear coordinate, δq = nuclear coordinate displacement/distortion in the excited state relative to the ground state (GS), and *E*_a_ = activation energy for relaxation from ^3^T_1_ to ^1^A_1_.

This leads us to the proposed picture in Figure 6b, where the ^3^T_1_ energy decreases from [Co­(phtpy)_2_]^3+^ to [Co­(dqp)_2_]^3+^ due to enhanced π-donation. However, the barrier (*E*_a_) for nonradiative relaxation from ^3^T_1_ to the singlet ground state (^1^A_1_) increases, as a result of larger force constants associated with the relevant potential energy surfaces, thereby extending the lifetime of the ^3^T_1_ state. In the case of the structurally comparable Cr^III^ complexes, the increased σ-donation and π-acceptance from [Cr­(tpy)_2_]^3+^ to [Cr­(dqp)_2_]^3+^ shifts the ligand-field dependent ^4^T_2_ state to higher energies (Figure 6a). This reduces the likelihood of reverse intersystem crossing from the luminescent ^2^E state to the ^4^T_2_ state, thereby prolonging the ^2^E lifetime.^48,49,57^ It seems plausible that intramolecular π–π-interactions in [Cr­(dqp)_2_]^3+^ could induce a similar rigidifying effect as proposed for [Co­(dqp)_2_]^3+^, contributing to the exceptionally slow deactivation rate of the luminescent ^2^E state reported previously.^57^

To conclude this section, the picture for Cr^III^ in Figure 6a has become a widely accepted model in the field and has been found applicable to several other d-block metal species,^48,49,52,53,55,57^ including Fe^II^,^76,86,87^ Ru^II^,^108–110,119,120^ Mn^I^, and Cr^0^.^38,40,68,69^ Optimization of the bite angles enhances the ligand field strength and prolongs excited-state lifetimes. The model for Co^III^ shown in Figure 6 is unconventional, demonstrating that optimization of bite angles with π-donor ligands decreases the ligand field strength. Nonetheless, prolonged excited-state lifetimes can still be achieved through the simultaneous optimization of rigidity.

### Energy Losses between Excitation and the Photoactive Excited State

Co^III^ polypyridines have emerged as exceptionally strong oxidizing agents in their photoactive ^3^T_1_ excited states, surpassing the oxidizing power of several benchmark Ir^III^ photocatalysts.^29^ This is particularly remarkable given the significant energy loss that occurs between photoexcitation and the photochemical reaction in this class of compounds.^91^ Co^III^ polypyridines typically absorb strongly only in the blue and UV regions of the spectrum, yet their ^3^T_1_ state retains just ∼1.35 eV of energy, indicating that more than 50% of the energy from a 400 nm excitation photon is not retained. In the following, we aim to gain a deeper insight into the initial energy dissipation processes within the [Co­(dqp)_2_]^3+^ complex.

Specifically, we performed femtosecond UV–vis TA spectroscopy in acetonitrile at room temperature and used spectroelectrochemistry to aid the assignment of the observed spectral features,^121^ recognizing the limitations of this approach.^122,123^ The TA spectra of [Co­(dqp)_2_]^3+^ (Figure 7, top) were analyzed in the spectral region from 390 to 490 nm using global fitting, revealing three species-associated spectra (SAS) (Figure S3). The SAS_2_ corresponds to the long-lived ^3^T_1_ state (8.3 ns); its time component was fixed as a constant for the global fit. The initially populated state (SAS_0_) is tentatively assigned to a ^1^ILCT state because a distinct ESA band at 460 nm matches with a broad absorption band in the differential absorption spectrum following ligand reduction in this complex (Figures S2a and S4). Unfortunately, the spectral features of the oxidized ligand are outside the spectral region accessible by the femtosecond TA experiment (Figures S2c and S3). We propose that this ^1^ILCT state undergoes vibrational cooling and an intersystem crossing to the triplet manifold (SAS_1_) within <1 ps. The spectral features of SAS_1_ are reminiscent of the lower-lying ^3^T_1_ state (SAS_2_), differing only in the somewhat more intense ESA band at 410 nm. A lifetime of 23 ps can be attributed to the internal conversion (IC) and vibrational cooling (VC) processes between the respective higher- and lower-lying ^3^MC excited states. This relatively slow process is phenomenologically reminiscent of Cu^I^ complexes,^104,124^ where prolonged ISC and IC lifetimes can result from a geometrical rearrangement in the excited-state landscape.

**Figure 7:**
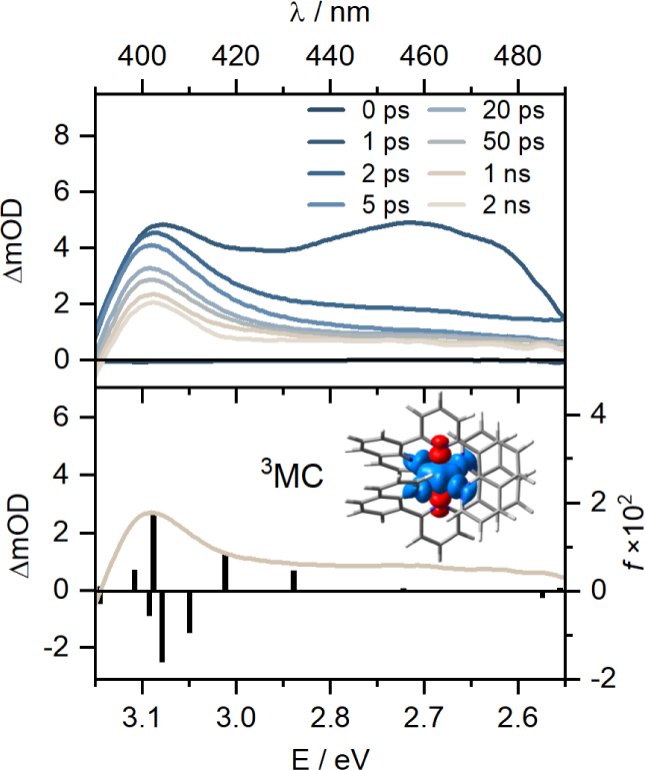
Top: UV–vis transient absorption spectra of [Co­(dqp)_2_]^3+^ in acetonitrile at 293 K, recorded at different delay times following excitation at 360 nm with pulses of ca. 190 fs duration; bottom: experimental TA spectrum, obtained at a delay time of 1 ns (solid line) and simulated TA spectrum of [Co­(dqp)_2_]^3+^ (black bars) at the TD-DFT level of theory with the spin density of the triplet state displayed.

While nanosecond excited-state lifetimes are typically required for diffusion-controlled bimolecular reactions,^125^ productive photoreactions can occur on the picosecond time scale when the photocatalyst and substrate molecules are preaggregated.^126,127,132^ A lifetime of 23 ps would, in principle, be sufficient for such processes. It is therefore conceivable that in appropriately preorganized systems such as pairs with anionic reaction partners,^128–131^ the 23 ps excited-state lifetime permits bimolecular (anti-Kasha) reactivity.^151^ This would represent an unconventional strategy for minimizing energy losses between excitation and the ensuing photochemical reaction.

To explore whether such a reaction can be observed, we attempted a photoinduced bimolecular triplet–triplet energy transfer (TTET) from the [Co­(dqp)_2_]^3+^ complex to perylene. Perylene has a triplet energy of 1.53 eV, significantly higher than the energy accessible from the relaxed ^3^T_1_ state of [Co­(dqp)_2_]^3+^ (1.26 eV) (Figure S9b).^133^ Thus, we were speculating that TTET could perhaps occur prior to internal conversion to the ^3^T_1_ state and its vibrational cooling (Figures S9b and 4d), accessed via preaggregation of the Co^III^ complex and perylene. The NMR titration experiment suggests the presence of the anticipated complex-substrate aggregates in the ground state (Figures S6–S8).^134^ The corresponding Benesi–Hildebrand plot yielded an association constant of *K*_a_ = 190 ± 50 M^–1^ in acetonitrile at room temperature.

Polypyridine Co^III^ complexes are known to form highly oxidizing species in their lowest triplet excited state (^3^T_1_),^29,46^ and [Co­(dqp)_2_]^3+^ is no exception, exhibiting an excited-state reduction potential of *E*_red_* = +1.59 V vs SCE. This excited-state redox potential is sufficient to oxidize perylene (*E*_ox_ = +0.85 V vs SCE) via a highly exergonic (Δ*G*_ET_^0^ = −0.74 eV) photoinduced bimolecular single-electron transfer (SET) process.^135^ Therefore, upon photoexcitation of [Co­(dqp)_2_]^3+^ in the presence of perylene, we anticipated to observe, using TA spectroscopy, the formation of both the perylene radical cation (perylene^+·^) via the SET mechanism from the fully relaxed ^3^T_1_ state of [Co­(dqp)_2_]^3+^ and the triplet perylene (^3^perylene) via the TTET mechanism potentially involving anti-Kasha behavior.^40,136,137^ Contrary to our initial expectations, we could only observe the formation of perylene^+·^ (Figure S9a) with its characteristic ESA signal at 540 nm and no evidence for an energy transfer from the higher triplet excited states of [Co­(dqp)_2_]^3+^.

In an attempt to shut down ordinary diffusion-controlled SET reactivity from the relaxed ^3^T_1_ excited state of [Co­(dqp)_2_]^3+^, as observed in the case of perylene discussed above, we aimed to explore the possibility of SET to more strongly oxidizing triplet excited states (with excited-state potentials exceeding 1.85 V vs SCE), accessible only through anti-Kasha reactivity of [Co­(dqp)_2_]^3+^. To this end, we investigated the possibility of photoinduced SET between [Co­(dqp)_2_]^3+^ and redox-challenging aromatic compounds such as durene (*E*_ox_ = +1.75 V vs SCE), 3,3′-dimethyl biphenyl (*E*_ox_ = +1.84 V vs SCE) and biphenyl (*E*_ox_ = +1.95 V vs SCE).^138,139^ However, in all cases, no evidence of radical cation formation was observed, suggesting that both thermodynamic and kinetic electron transfer remained unfavorable. Direct laser spectroscopic evidence for photoreactivity from higher excited states of [Co­(dqp)_2_]^3+^ is therefore difficult to provide at present, but could perhaps be found more indirectly in long-term light irradiation experiments, such as those carried out in classical synthetic photoredox catalysis.

The inherent photostability of [Co­(dqp)_2_]^3+^ in deaerated acetonitrile at room temperature is promising for potential photocatalytic applications, as the photodegradation quantum yield under these conditions is remarkably low, only 0.003% (Figure S5). To put this value into perspective, the benchmark complex [Ru­(bpy)_3_]^2+^ has been reported to exhibit a significantly higher photodegradation quantum yield under comparable conditions.^140^ Furthermore, among the Co^III^ complexes, [Co­(ppy)_3_] and [Co­(CN)_6_]^3–^ were previously shown to undergo photodissociation from the ^3^T_1_ excited state.^97,141^ The [Co­(ppy)_3_] complex undergoes significant Jahn–Teller distortion in an electronically excited state, leading to its ∼9 ps deactivation via Co–C_ph_ bond rupture.^141^ The photoaquation reaction of [Co­(CN)_6_]^3–^ occurs in a similar fashion.^97^

## Conclusions

Our work reveals two counterintuitive insights into the molecular design principles and photophysical behavior of d-metal complexes. First, the widely accepted paradigm that optimizing the coordination bite angle strengthens the ligand field is not supported in this study.^48,49,52,56,57,61,77,86,87^ Instead, we find that such optimization can actually weaken the ligand field when the ligands function primarily as π-donors.^99^ Second, the well-known “energy gap law,”^93^ which states that nonradiative excited-state relaxation accelerates as the excited-state energy decreases, does not hold in the case investigated here. This is because intramolecular rigidification exerts a counteracting influence that can significantly prolong excited-state lifetimes. Both of these key findings are, in principle, predictable based on ligand field theory (Figure 2) and simple considerations of the relevant potential well diagrams (Figure 6).^105^ Although π-donor ligands in photoactive metal complexes have been receiving growing attention,^53,73,100,122,123,142–147^ and the concept of cooperative rigidity among ligands coordinated to the same metal center is becoming increasingly well established,^40,104–106,148,149^ the direct comparison between [Co­(phtpy)_2_]^3+^ and [Co­(dqp)_2_]^3+^ presented in this work provides rare and fundamental insight into both of these aspects.

Our study further suggests the potential for conducting bimolecular photochemistry from higher electronically excited states. This approach could help minimize energy losses between light absorption and photochemical energy storage, especially in Co^III^ polypyridines, which absorb in the blue spectral range but have low-lying excited states that store significantly less energy than those of traditional Ru^II^ polypyridines or cyclometalated Ir^III^ complexes.

The specific insights gained from our study can guide the future designs of photoactive complexes based on both abundant first-row and noble or non-noble second- and third-row transition metals, as metal-centered excited states and their deactivation pathways universally play a critical role in determining the photophysical and photochemical properties.^150^ The broader concept that a ligand may act as a π-acceptor with one metal but as a π-donor with another could become increasingly relevant as unconventional metal oxidation states are more widely explored, and novel ligand frameworks continue to emerge. Similar effects to those observed in this study may therefore be anticipated in yet-to-be-discovered metal complexes, where a delicate interplay between metal and ligand orbital energies, orbital overlap, and intramolecular π–π interactions shapes the excited-state energy landscape that ultimately governs luminescence properties and photochemical behavior.

## Supplementary Material



## Notes

Experimental procedures, materials and methods; synthesis and characterization of complexes, single crystal X-ray data, NMR and HR-ESI mass spectra, photophysical and electrochemical data as well as computational details and in-depth quantum chemical results (PDF)
