# Supplementary material for: Structural Control of Metal-Centered Excited States in Cobalt(III) Complexes via Bite Angle and π–π Interactions
Source: J Am Chem Soc. 2025 Jul 30;147(32):29444–56. doi: 10.1021/jacs.5c09616 (PMC12356593; doi:10.1021/jacs.5c09616)
Supplement: Supplementary file 1 [file ja5c09616_si_001.pdf]

Supporting Information

Structural Control of Metal-Centered Excited States in Cobalt(III) Complexes via Bite Angle and π-π Interactions

Polina Yaltseva[a], Tamar Maisuradze[b], Alessandro Prescimone[a], Stephan Kupfer[b]* and Oliver S. Wenger[a]*

[a] Department of Chemistry, University of Basel, St. Johanns-Ring 19, 4056 Basel, Switzerland

[b] Institute of Physical Chemistry, Friedrich-Schiller-Universität Jena, Helmholtzweg 4, 07743 Jena, Germany

Table of contents

Methods and experimental procedures S2

Synthesis of $[Co(dqp)_2](PF_6)_3$ S4

Femtosecond UV-vis transient absorption S7

Investigation of aggregation between $[Co(dqp)_2]^{3+}$ and perylene in solution via ^{1}H NMR titration ...S11

Evaluating the possibility of triplet-triplet energy transfer or single electron transfer between $[Co(dqp)_2]^{3+}$ and perylene S14

XRD data S16

NMR spectra S18

Mass spectra S22

Quantum chemistry S23

Computational details S23

Computational results S24

References S49

Methods and experimental procedures

All chemicals for synthesis were purchased from commercial suppliers in reagent grade and used without any further purification. 4'-phenyl-2,2':6',2"-terpyridine (phtpy), 2,6-di(quinolin-8-yl)pyridyl (dqp) and $[Co(phtpy)_2](PF_6)_3$ were synthesized according to literature procedures.[1,2] Solvents for synthesis and spectroscopy were purchased in extra dry (>99.5% purity) and HPLC grades, solvents for synthesis were additionally degassed via freeze-pump-thaw cycles.

NMR spectra were recorded on Bruker Avance III instruments with 250, 400 or 500 MHz proton frequency. Deuterated solvents were purchased from Eurisotop (Cambridge Isotope Laboratories). Chemical shifts were reported in δ values in ppm and were referenced to the solvent residue peak.[3]

Cyclic voltammetry was conducted under argon atmosphere in an electrochemical cell containing a glassy carbon disk as the working electrode, a silver wire as the counter electrode, and a saturated calomel electrode (SCE) as the reference electrode. Potentiostat Versastat 3-200 from Princeton Applied Research was used.

Steady-state absorption spectroscopy was measured on a Varian Cary 5000 UV-vis-NIR instrument, using optical glass cuvettes with 1 cm and 10 cm path length. Deconvolution analysis of obtained UV-Vis absorption spectra was performed using Origin software.

UV-vis transient absorption measurements on the picosecond timescale were carried out with a TRASS instrument from Hamamatsu and a mode-locked picosecond Nd:YVO_4/YAG laser from Ekspla (PL2251B-20-SH/TH/FH with PRETRIG option, ca. 30 ps pulse width) as excitation light source. The frequency-tripled (355 nm) output with power of 6 mJ per pulse was used for the experiments. UV-vis transient absorption measurements on the femtosecond timescale were carried out with a HARPIA-TA instrument (Light Conversion). Excitation light was generated by a PHAROS laser (Light Conversion, Yb:KGW laser, source wavelength of 1030 nm, pulse width of ca. 190 fs, pulse energy ~0.2 mJ), and the actual pump light wavelength was generated by an OPA ORPHEUS (Light Conversion, used ~ 90% of fundamental pulse). The probe light was generated by a sapphire (5 mm thickness; ~10% of the fundamental pulse were used to generate a white light super-continuum). UV-vis transient absorption measurements on the nanosecond timescale were carried out on an LP920-KS instrument from Edinburgh Instruments. A frequency-tripled Nd:YAG laser (Quantel Brilliant, ca. 10 ns pulse width) equipped with OPO from Opotek and a beam expander (GBE02-A from Thorlabs) in the beam path was used for excitation at 450 nm (pulse energy of ~15 mJ). An iCCD camera from Andor was used to detect transient absorption spectra.

Differential UV-vis absorption spectra of electrochemically generated species were measured using Ocean HDX UV-vis spectrometer from Ocean Optics. Setup consisted of a 1 mm quartz cuvette, equipped with a platinum grid as the working electrode, a platinum wire as the counter electrode, and a saturated calomel electrode (SCE) as the reference electrode.

Elemental analysis was carried out by Sylvie Mittelheisser on a Vario Micro Cube instrument (University of Basel, Department of Chemistry). Electrospray ionization high resolution mass spectra (ESI-HRMS) were recorded by Dr. Michael Pfeffer (University of Basel, Department of Chemistry) on a Bruker maxis 4G ESI-Q-TOF instrument.

Preparative HPLC-MS was performed on a Shimadzu LCMS-2020 single quadrupole instrument with a Diode Array Detector. An Agilent ZORBAX XDB-C18 column (7.0 µm, 21.2 × 250 mm); solvent system A (H_2O, 0.1% formic acid) and B (acetonitrile with 0.1% formic acid) were used for the separation. Injections were made in 99% A, held at 99% A for 5 minutes, ramped to 60% B over 15 minutes, then to 99% B over 10 min. 99% B was held for 5 min, and then returned to starting conditions over 1 min and allowed to re-equilibrate for 4 minutes with a 20 mL/min constant flow rate.

For photostability experiments, an Ocean HDX UV-vis spectrometer from Ocean Optics was used with a continuous-wave (cw) laser from Roithner Lasertechnik emitting at 405 nm (2 W/cm^2). Spectroscopic experiments with that cw-laser as a light source were carried out at 293 K using self-built cuvette holders with temperature control.

Synthesis of $[Co(dqp)_2](PF_6)_3$

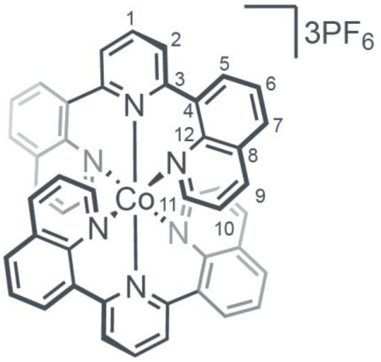

2,6-Di(quinolin-8-yl)pyridyl (dqp) ligand (550 mg, 1.65 mmol, 2.0 eq.) was suspended in 20 mL CH_3CN. To the suspension, a solution of $CoCl_2 \cdot 6H_2O$ (196 mg, 825 µmol, 1.0 eq.) in 8 mL H_2O was added dropwise, resulting in clear dark red solution. After the addition, the mixture was heated to 70 ºC for 1.5 hours. The mixture was cooled down to room temperature and bromine (110 µL, 2.06 mmol, 2.5 eq.) in 3 mL of H_2O was added dropwise. The resulting solution was stirred for 16 hours under exclusion of light. Saturated aqueous KPF_6 was added, and the mixture was stirred for 30 min and 50 mL of water was added. The bright orange precipitate was filtered off and washed with excess H_2O, cold EtOH and finally Et_2O. The target compound was isolated as bright orange solid (520 mg, 0.512 mmol, 62.1%).

^{1}H NMR (CD_3CN, 298 K, 500 MHz) δ/ppm: 8.52 (dd, J = 8.00 Hz, 1.27 Hz, 2H, **9**), 8.47 (t, J = 7.91 Hz, 1H, **1**), 8.11 (m, 4H, **2** and **11**), 8.04 (dd, J = 7.44 Hz, 1.31 Hz, 2H, **7**), 7.94 (dd, J = 8.29 Hz, 1.20 Hz, 2H, **5**), 7.70 (dd, J = 8.27 Hz, 7.44 Hz, 2H, **6**), 7.43 (dd, J = 8.00 Hz, 5.62 Hz, 2H, **10**).

^{13}C{^{1}H} NMR (CD_3CN, 298 K, 126 MHz) δ/ppm: 163.1 (**11**), 155.5 (**3**), 144.8 (**8**), 144.3 (**9**), 143.4 (**1**), 134.1 (**7**), 133.7 (**5**), 130.6 (**2**), 129.1 (**4**), 128.9 (**6**), 127.8 (**12**), 124.9 (**10**).

HRMS (positive mode): calc. for $[C_{46}H_{30}CoN_6]^{3+}$ 241.7282 m/z. Found: 241.7285 m/z.

Anal. calcd. for $C_{46}H_{30}CoF_{18}N_6P_3 \cdot 2(H_2O)$ (%): C, 46.17; H, 2.86; N, 7.02. Found (%): C, 45.90; H, 2.77; N, 7.02.

Electrochemistry and spectroelectrochemistry

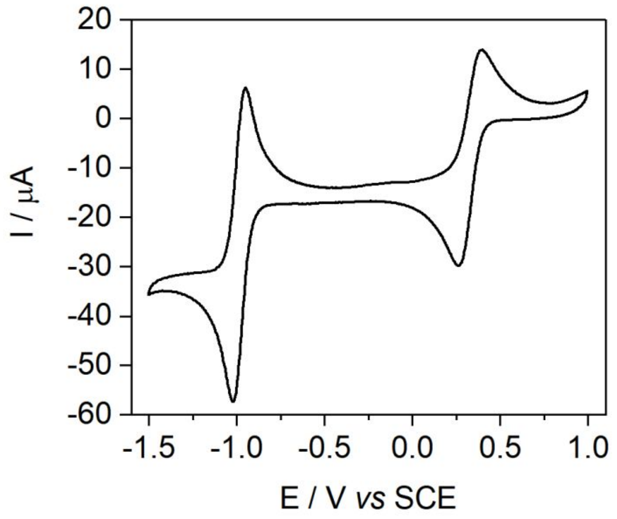

Figure S1. Cyclic voltammogram of 1 mM $[Co(dqp)_2](PF_6)_3$ in dry and deaerated acetonitrile with 0.1 M $TBAPF_6$ as the supporting electrolyte measured against an SCE reference. A glassy carbon disk was used as the working electrode, and a silver wire served as the counter electrode.

The second reversible reduction event in $[Co(dqp)_2]^{3+}$ at −0.99 V *vs.* SCE corresponds to the ligand-based reduction. Compared to $[Co(phtpy)_2]^{3+}$ (ligand-based reduction at −0.75 V *vs.* SCE[4]) this process is shifted cathodically, in agreement with higher electron density on the dqp ligand compared to phtpy.

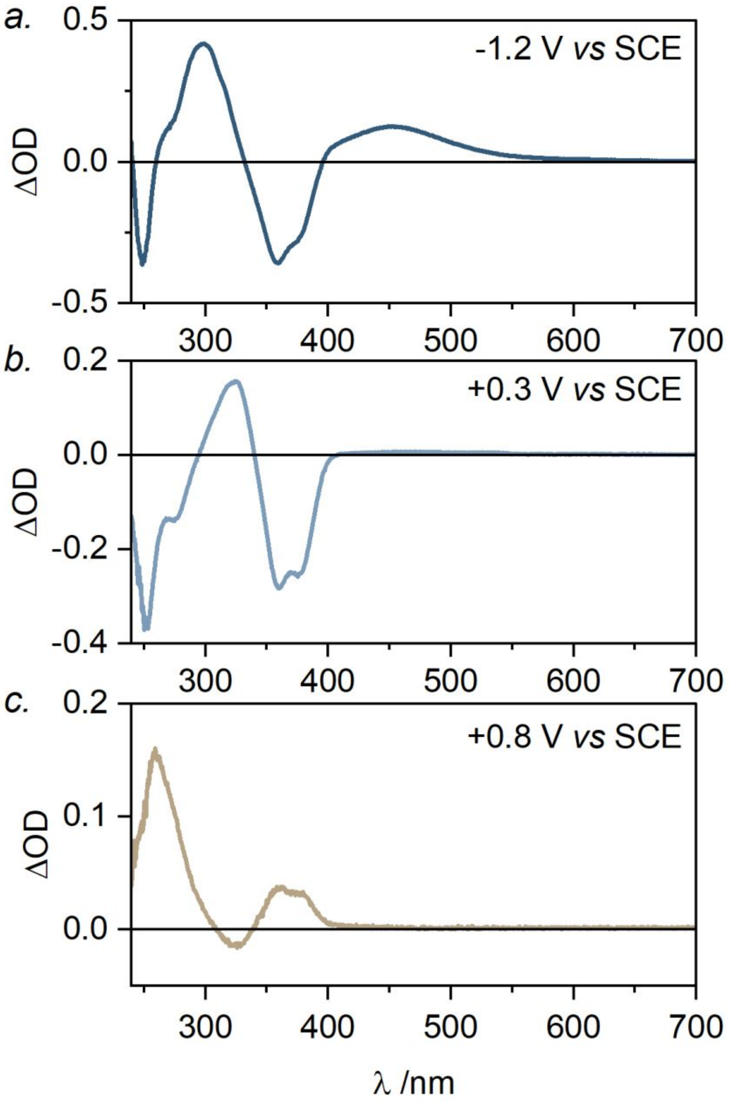

Figure S2. ***a***. UV-vis differential (ground-state) absorption spectrum of $[Co(dqp)_2]^{3+}$ in deaerated CH_3CN at room temperature with -1.2 V *vs* SCE potential applied, corresponding to a ligand-based $L^0/L^{-\cdot}$ reduction process. ***b***. UV-vis differential (ground-state) absorption spectrum of $[Co(dqp)_2]^{3+}$ in deaerated CH_3CN at room temperature with +0.3 V *vs* SCE potential applied, corresponding to a metal-based Co^{II}/Co^{III} reduction process; ***c.*** UV-vis differential (ground-state) absorption spectrum of $[Co(dqp)_2]^{3+}$ in deaerated CH_3CN at room temperature with +0.8 V *vs* SCE potential applied, corresponding to a ligand-based oxidation process $L^{+\cdot}/L^0$.

Femtosecond UV-vis transient absorption

We have chosen $[Co(dqp)_2]^{3+}$ exclusively for advanced spectroscopic investigation, as we have speculated that similar to the literature-known examples of Cu^I and Fe^{II} complexes,[5,6] cooperative rigidity (facilitated by π-π stacking) in this complex could potentially influence both the excited-state lifetimes of the lowest excited state ($^3T_1$) and the internal conversion rates between higher excited states and the lowest excited state.

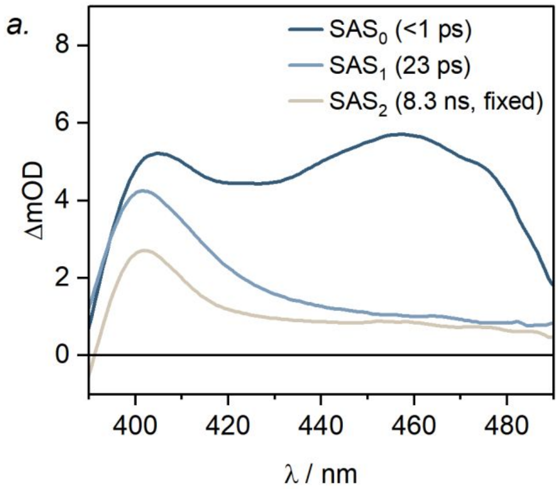

Figure S3. Result of the global fit analysis of femtosecond UV-vis TA spectra of $[Co(dqp)_2]^{3+}$ on the data in Figure 7 (top); for the fitting a sequential excited state population model was used. Obtained species associated spectra (SAS) and corresponding excited state lifetimes are indicated in the inset.

Table S1. Global fit analysis of the transient absorption measurements (Figure 7). Species-associated spectra (SAS_0-SAS_2) corresponding to the time components τ_0-τ_2 are shown in Figure S3.

**Complex**	**τ_0 / ps**	**τ_1 / ps**	**τ_2 / ps**
**$[Co(dqp)_2]^{3+}$**	0.7±0.4	22.5±8.2	8300 (fixed)

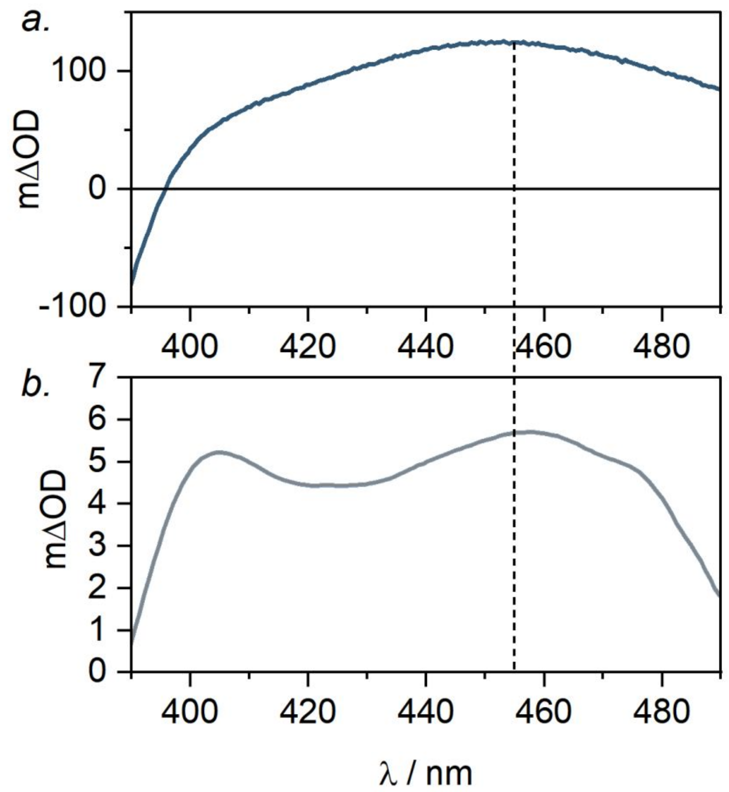

Figure S4. ***a***. UV-vis differential (ground-state) absorption spectrum of $[Co(dqp)_2]^{3+}$ (Figure S2a) in deaerated CH_3CN at room temperature with -1.2 V *vs* SCE potential applied, corresponding to a ligand-based $L^0/L^{-\cdot}$ reduction process; ***b.*** SAS_0 (Figure S3) obtained for the component corresponding to $\tau_0 < 1$ ps from the femtosecond TA spectra of $[Co(dqp)_2]^{3+}$ (Figure 7).

Photostability

Deaerated acetonitrile solution of the sample in quartz cuvette was irradiated with a 405 nm continuous-wave laser of 100 mW power output. The temperature was kept constant at 293 K. After irradiation start, UV-vis absorption spectra were recorded every 2 min within the course of 2 hours. The optical density at 380 nm was plotted as a function of irradiation time. Photodegradation quantum yield was calculated according to the following expression (eq. S1) at the time point of 10% decrease in the initial optical density at the monitored wavelength.

$$\varphi_{degr} = \frac{n_{decomp}}{n_{abs}} \cdot 100\% \qquad \textbf{S1}$$

In eq.S1 n_{decomp} is the number of the decomposed molecules of the target compound and n_{abs} is the number of the photons initially absorbed by the sample, calculated according to eq. S2 and S3 respectively.

$$n_{decomp} = 0.9 \cdot V \cdot c_0 \qquad \textbf{S2}$$

$$n_{abs} = (1 - T) \cdot \frac{p_{laser} \cdot t}{E_{laser} \cdot N_A} \qquad \textbf{S3}$$

In eq. S2 and S3:

T - transmittance at the irradiation wavelength, $T = 10^{-A}$;

c_0 - initial molar concentration of the target compound [M], $c_0 = \frac{A_0}{\varepsilon \cdot l}$;

V - volume of the solution [l];

p_{laser} – laser power output at 405 nm [W];

t – irradiation time, at which 10% of the target molecules decomposed [s];

E_{laser} – energy of a photon at 405 nm [J], $4.89 \cdot 10^{-19}$ J;

N_A – Avogadro's constant [mol^{-1}], $6.022 \cdot 10^{23}$ mol^{-1}.

Table S2. Calculation of the relevant experimental parameters and photodegradation quantum yields, (φ_{degr}) following eq. S1-S3.

Sample	c_0 [μM]	t [s]	n_{decomp} [mol]	n_{abs} [mol]	φ_{degr} [%]
**[Co(dqp)$_2$]$^{3+}$**	27.5	11400	$9.9 \cdot 10^{-8}$	$3.8 \cdot 10^{-3}$	0.003

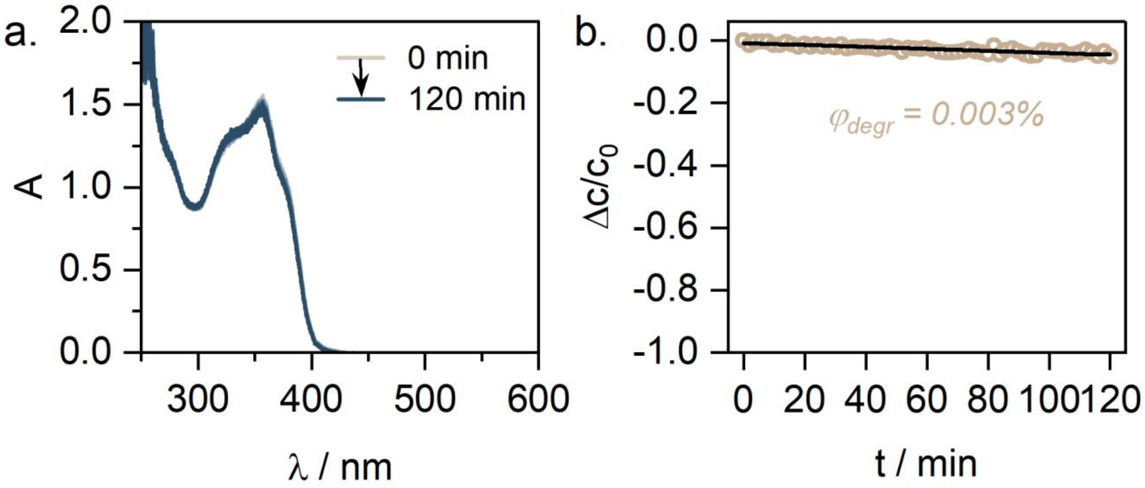

Figure S5. (a) Absorption spectra of $[Co(dqp)_2]^{3+}$ recorded every 2 min (see inset for the graph color coding) upon irradiation with a 100 mW 405 nm continuous-wave laser. The optical density at the excitation wavelength was 0.06. (b) Photostability profile of $[Co(dqp)_2]^{3+}$ irradiated with a 405 nm cw laser (2 W/cm^2) in deaerated acetonitrile at room temperature, φ_{degr} reported at A/A_0=0.9. Concentration changes $\Delta c/c_0$ were calculated monitoring optical density at 380 nm.

Investigation of aggregation between $[Co(dqp)_2]^{3+}$ and perylene in solution via ^1H NMR titration

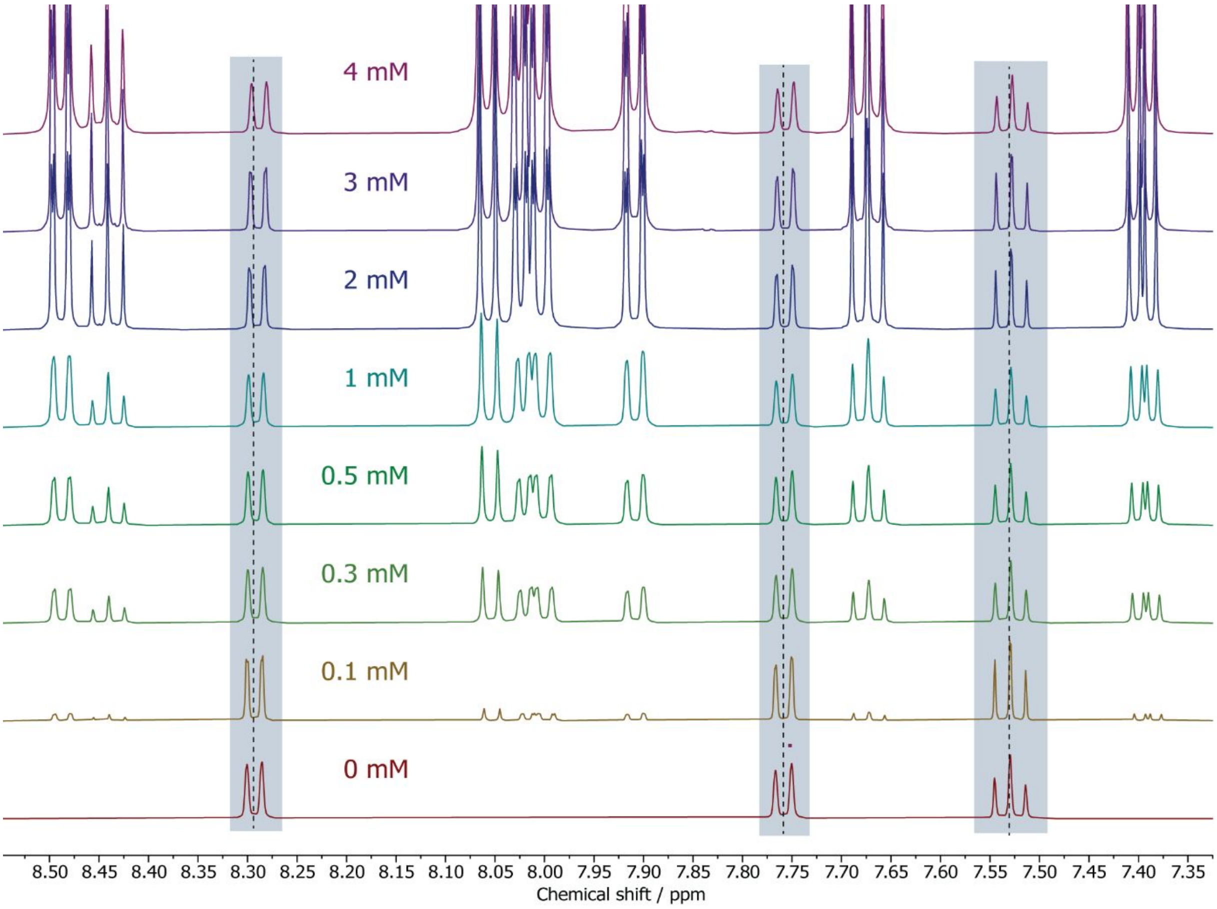

Figure S6. Titration experiment: ^1H NMR spectra of 1mM perylene and 0-4 mM $[Co(dqp)_2]^{3+}$ solutions in CD_3CN. Signals corresponding to perylene are marked in grey color.

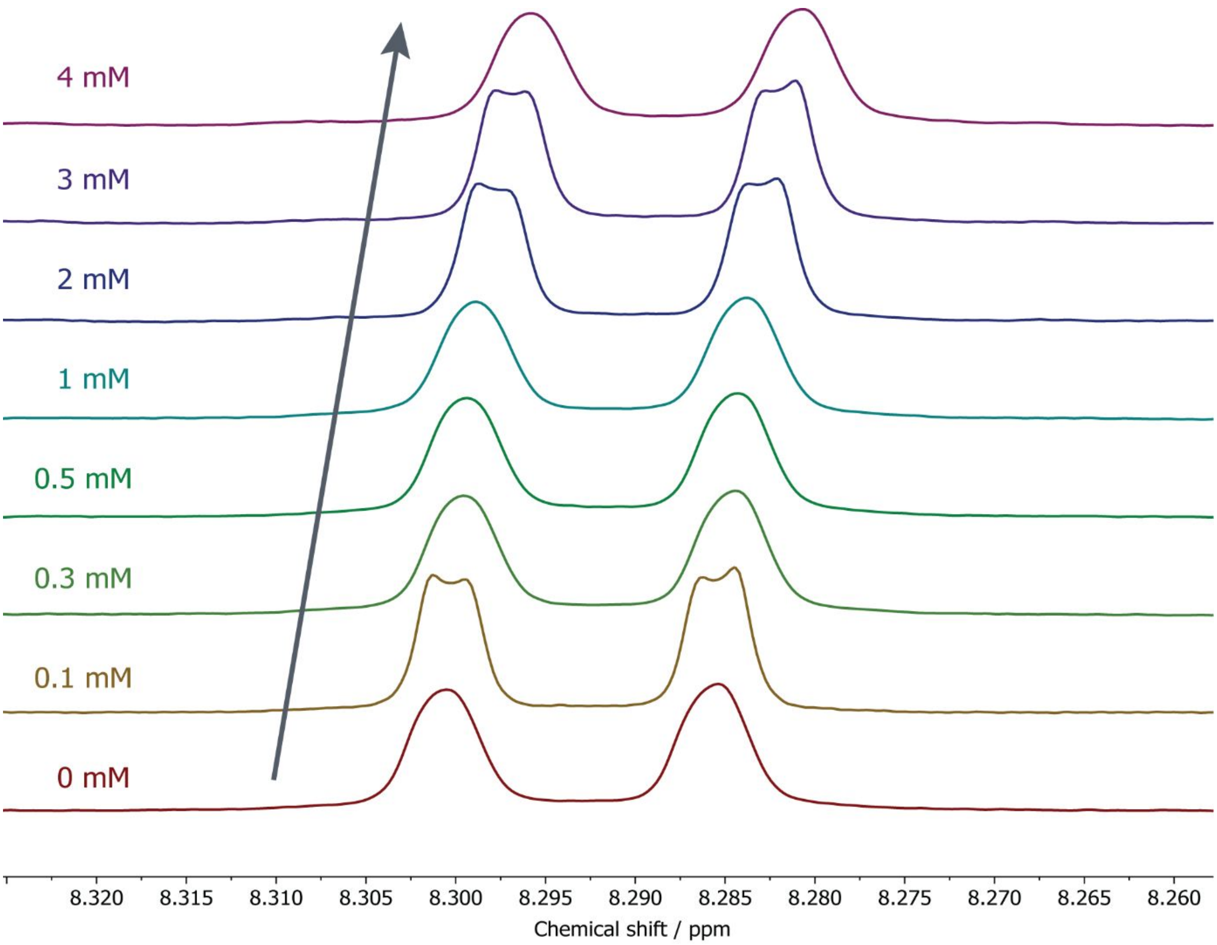

Figure S7. ^{1}H NMR spectra of 1mM perylene and 0.1-4 mM $[Co(dqp)_2]^{3+}$ solutions in CD_3CN showing the signal of one of the perylene protons.

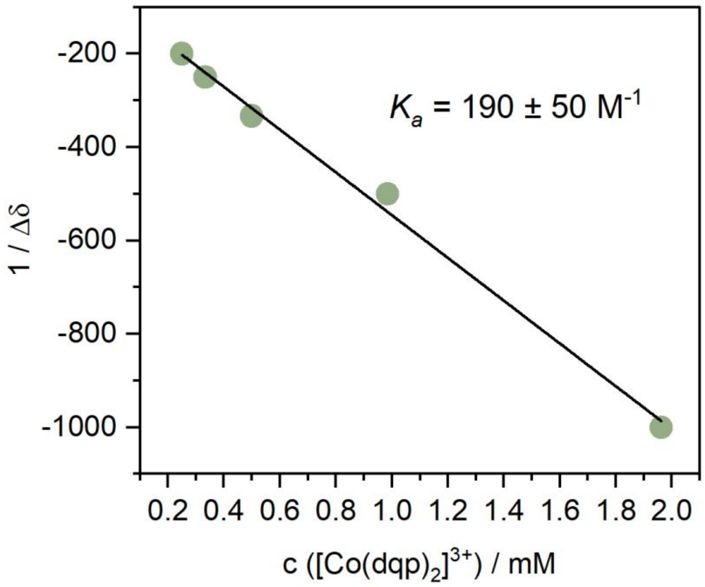

Figure S8. Benesi-Hildebrand plot for the NMR titration of 1mM perylene with 0.1-4 mM $[Co(dqp)_2]^{3+}$. $\Delta\delta = \delta - \delta_0$ corresponds to the changes in the chemical shift of the perylene proton at δ_0 =8.293 ppm (Figure S7). The slope of −458 M^{-1}ppm^{-1} and the intercept of −87 ppm^{-1} allow to obtain the association constant of K_a = 190 ± 50 M^{-1}, according to the equation **S4** with the assumption of 1:1 association.[7]

$$\frac{1}{\Delta\delta} = \frac{1}{K_a \Delta\delta_{max}} \cdot \frac{1}{[c]} + \frac{1}{\Delta\delta_{max}} \quad \textbf{S4}$$

In eq. S4[7]:

$\Delta\delta$ - change in chemical shift upon addition of the complex relative to the non-associated substrate [ppm];

K_a - association constant [M^{-1}];

$\Delta\delta_{max}$ - difference in chemical shifts between the non-associated substrate and the aggregate [ppm];

[c] - concentration of complex (titrant) [M].

Evaluating the possibility of triplet-triplet energy transfer or single electron transfer between $[Co(dqp)_2]^{3+}$ and perylene

We have attempted a photoinduced bimolecular triplet-triplet energy transfer (TTET) from the $[Co(dqp)_2]^{3+}$ complex to perylene. Perylene has a triplet energy of 1.53 eV, significantly higher than the energy accessible from the relaxed $^3T_1$ state of $[Co(dqp)_2]^{3+}$ (1.26 eV) (Figure S9b).[8] Thus, we were speculating that TTET could perhaps occur prior to internal conversion to the $^3T_1$ state and its vibrational cooling (Figure 4d, S9b), accessed via pre-aggregation of the Co^{III} complex and perylene.

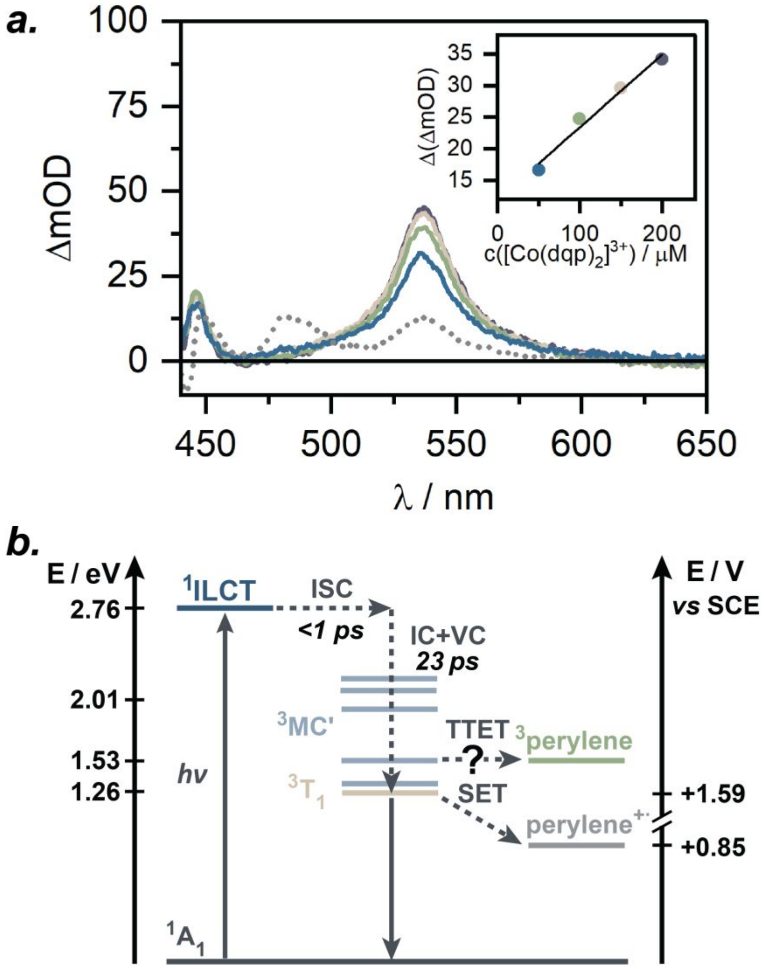

Figure S9. a. UV-vis TA spectra of 1 mM perylene with the additives of 50-200 µM $[Co(dqp)_2]^{3+}$ in deaerated acetonitrile (for the color coding see the inset) following the excitation at 450 nm with nanosecond pulses, recorded with a 1 µs delay and time-integrated over 10 µs; inset: Stern-Volmer-like plot, showing the dependency of the corrected (relative to the reference) perylene$^{+\cdot}$ signal intensity at 540 nm and $[Co(dqp)_2]^{3+}$ concentration. The reference TA spectrum of 1 mM perylene in deaerated acetonitrile shown in a dashed line was used as a baseline reference and all intensity values shown in the inset were corrected in regard to it. b. Jablonski diagram depicting the electronically excited states of $[Co(dqp)_2]^{3+}$ and possible triplet-triplet energy transfer (TTET) or single electron transfer (SET) to perylene.

$[Co(dqp)_2]^{3+}$ exhibits an excited state reduction potential of $E_{red}^* = +1.59$ V *vs.* SCE, sufficient to oxidize perylene ($E_{ox} = +0.85$ V *vs.* SCE) via a highly exergonic ($\Delta G_{ET}^0 = -0.74$ eV) photoinduced bimolecular single electron transfer (SET) process.[9] Therefore, upon photoexcitation of $[Co(dqp)_2]^{3+}$ in the presence of perylene, we anticipated to observe, using TA spectroscopy, the formation of both the perylene radical cation (perylene$^{+\cdot}$) via the SET mechanism from the fully relaxed $^3T_1$ state of $[Co(dqp)_2]^{3+}$ and the triplet perylene (^{3}perylene) via the TTET mechanism potentially involving anti-Kasha behavior. The signature ESA signals of perylene$^{+\cdot}$ and ^{3}perylene species are expected at 540 nm and ~500 nm respectively according to the literature.[9–11]

Upon photoexcitation at 450 nm with nanosecond pulses of perylene solutions in acetonitrile, containing increasing concentrations of $[Co(dqp)_2]^{3+}$, we observed the characteristic ESA signal at 540 nm, corresponding to the perylene$^{+\cdot}$ (Figure S9a). Contrary to our initial expectations, no signal corresponding to the formation of ^{3}perylene was observed, indicating the absence of an energy transfer from the higher triplet excited states of $[Co(dqp)_2]^{3+}$. The linear, pseudo Stern-Volmer, dependence of perylene$^{+\cdot}$ ESA signal intensity on the concentration of the complex supports the assumption that the observed signal enhancement arises from the reductive quenching of the $^3T_1$ state of $[Co(dqp)_2]^{3+}$ by perylene. Presumably, both diffusion-controlled SET from the lowest triplet excited state of $[Co(dqp)_2]^{3+}$, and aggregation-based SET involving higher triplet states can contribute to the formation of observed perylene$^{+\cdot}$, although the latter is speculative. The direct excitation of perylene solution at 450 nm shows a minor perylene$^{+\cdot}$ TA signal, which could be due to the trace impurities present in commercially available perylene (Figure S9a, dashed line).

XRD data

Table S3. Crystal data and structure refinement.

Identification code	YAL-200_150K
CCDC number	2422208
Empirical formula	$C_{50}H_{36}CoF_{18}N_8P_3$
Formula weight	1242.71
Temperature/K	150
Crystal system	monoclinic
Space group	$P2_1/n$
a/Å	11.5258(2)
b/Å	30.7411(6)
c/Å	14.1855(2)
α/°	90
β/°	104.8390(10)
γ/°	90
Volume/Å^3	4858.52(15)
Z	4
ρ_{calc}g/cm^3	1.699
μ/mm^{-1}	3.233
F(000)	2504.0
Crystal size/mm^3	0.2 × 0.14 × 0.1
Radiation	GaKα (λ = 1.34143)
2Θ range for data collection/°	6.14 to 111.338
Index ranges	$-13 \leq h \leq 14$, $-36 \leq k \leq 37$, $-17 \leq l \leq 10$
Reflections collected	75464
Independent reflections	9402 [R_{int} = 0.0837, R_{sigma} = 0.0392]
Data/restraints/parameters	9402/0/724
Goodness-of-fit on F^2	1.010
Final R indexes [I>=2σ (I)]	R_1 = 0.0460, wR_2 = 0.1077
Final R indexes [all data]	R_1 = 0.0670, wR_2 = 0.1191
Largest diff. peak/hole / e Å^{-3}	0.54/-0.65

$Co(dqp)_2(PF_6)_3$ was recrystallized from a mixture of MeCN and Et_2O by solvent layering. A suitable crystal was selected and mounted on a MITIGEN holder in perfluoroether oil on a STOE STADIVARI diffractometer. The crystal was kept at 150 K during data collection. Using Olex2[12], the structure was solved with the SHELXT[13] structure solution program using Intrinsic Phasing and refined with the SHELXL[14] refinement package using Least Squares minimization.

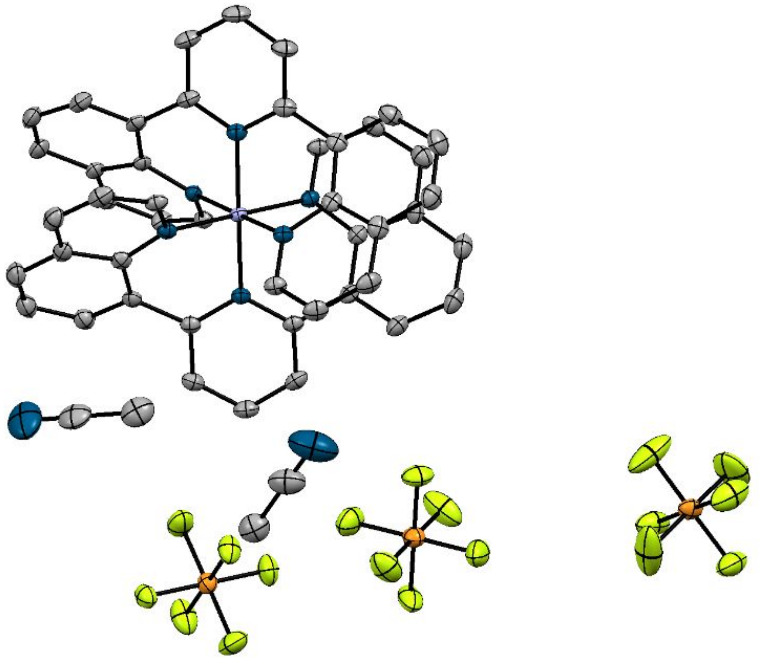

Figure S10. X–ray crystal structure of $Co(dqp)_2(PF_6)_3 \cdot 2CH_3CN$ with thermal ellipsoids at 50% probability. Hydrogen atoms are omitted for clarity.

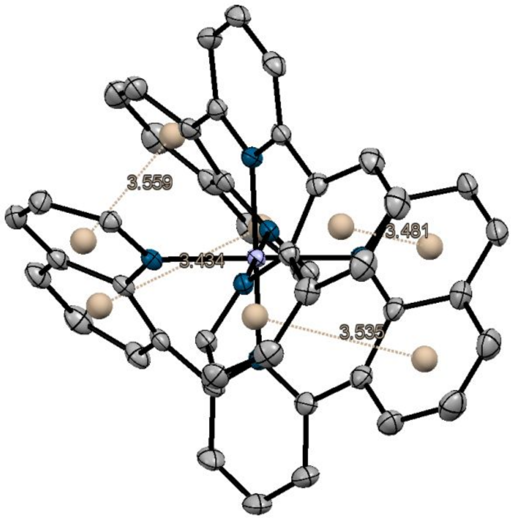

Figure S11. X-ray crystal structure of $[Co(dqp)_2]^{3+}$ with the indication of the π-π interactions, relevant centroids and the distances between them are shown. Hydrogen atoms, counter ions and solvent molecules are omitted for clarity. Thermal ellipsoids at 50% probability.

NMR spectra

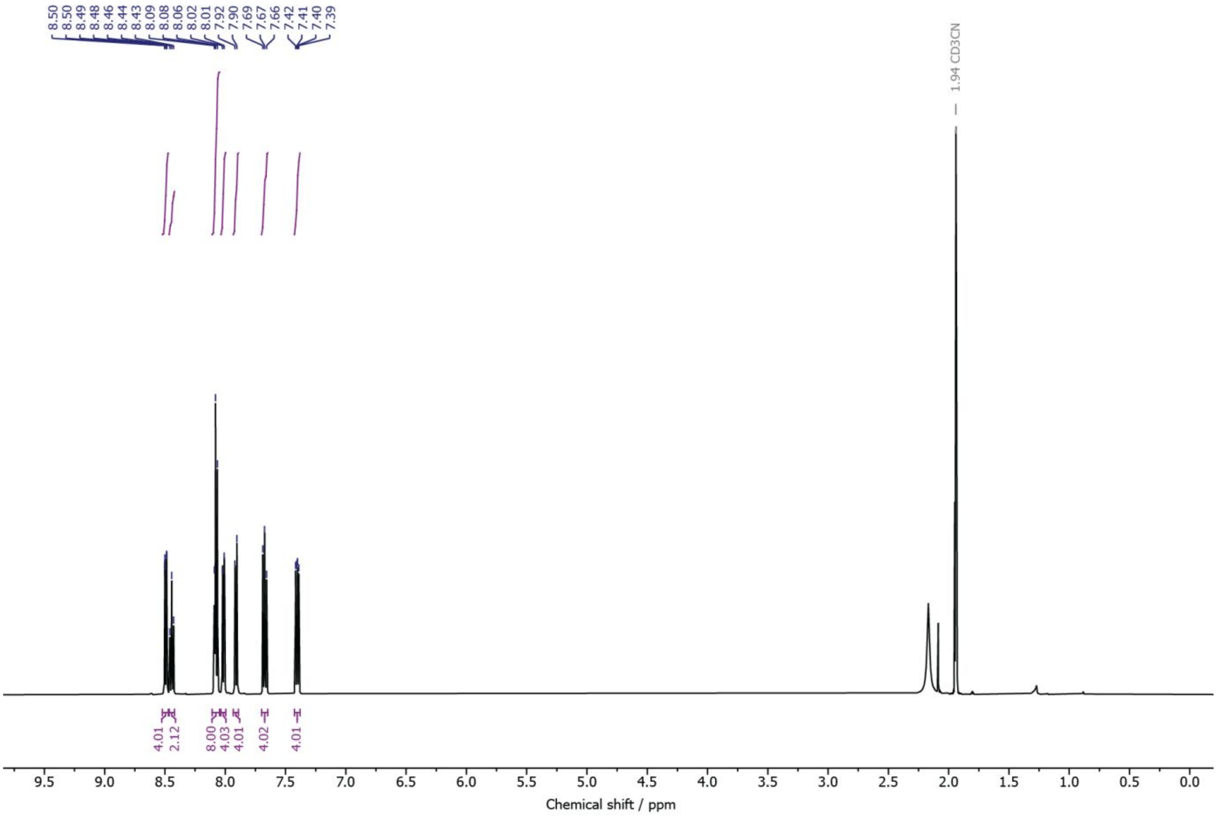

Figure S12. ^{1}H NMR (500 MHz, 298 K) spectrum of $[Co(dqp)_2](PF_6)_3$ in CD_3CN.

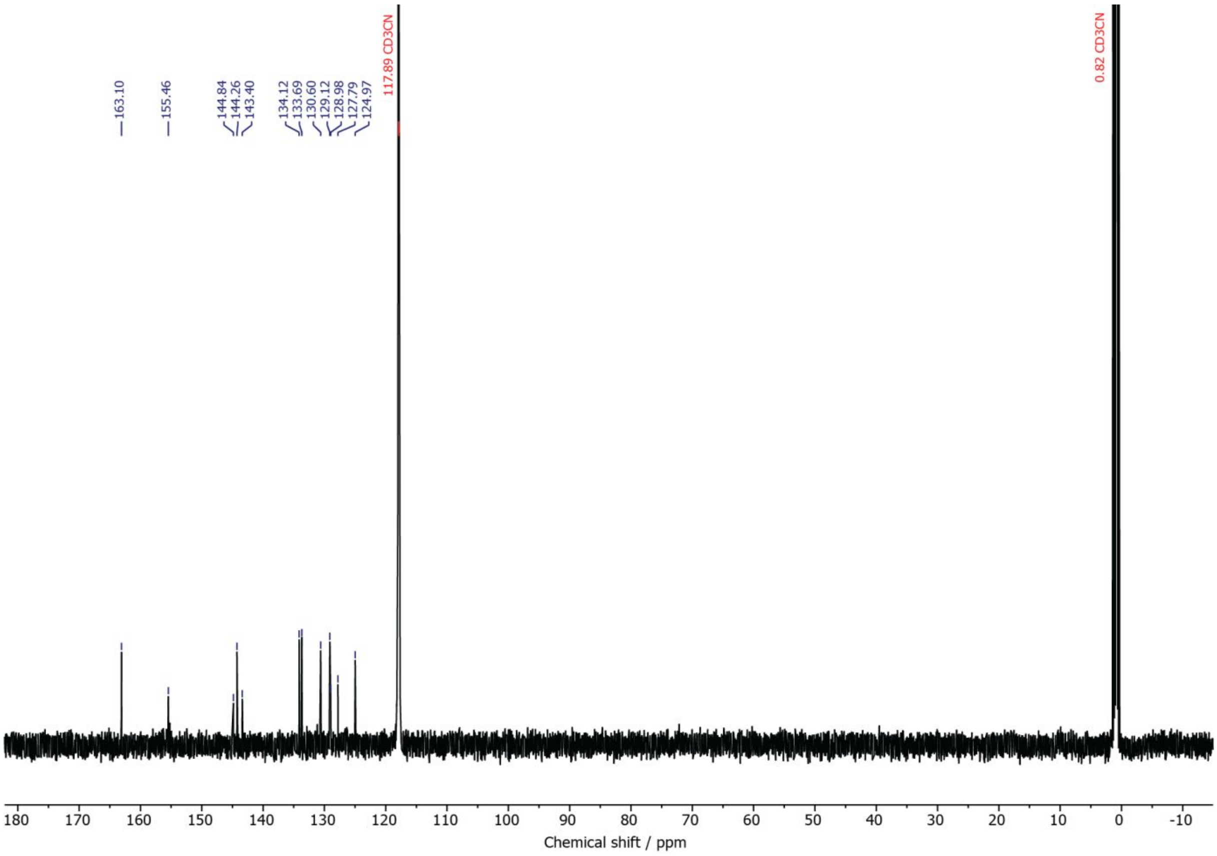

Figure S13. $^{13}C\{^1H\}$ NMR spectrum of $[Co(dqp)_2](PF_6)_3$ in CD_3CN.

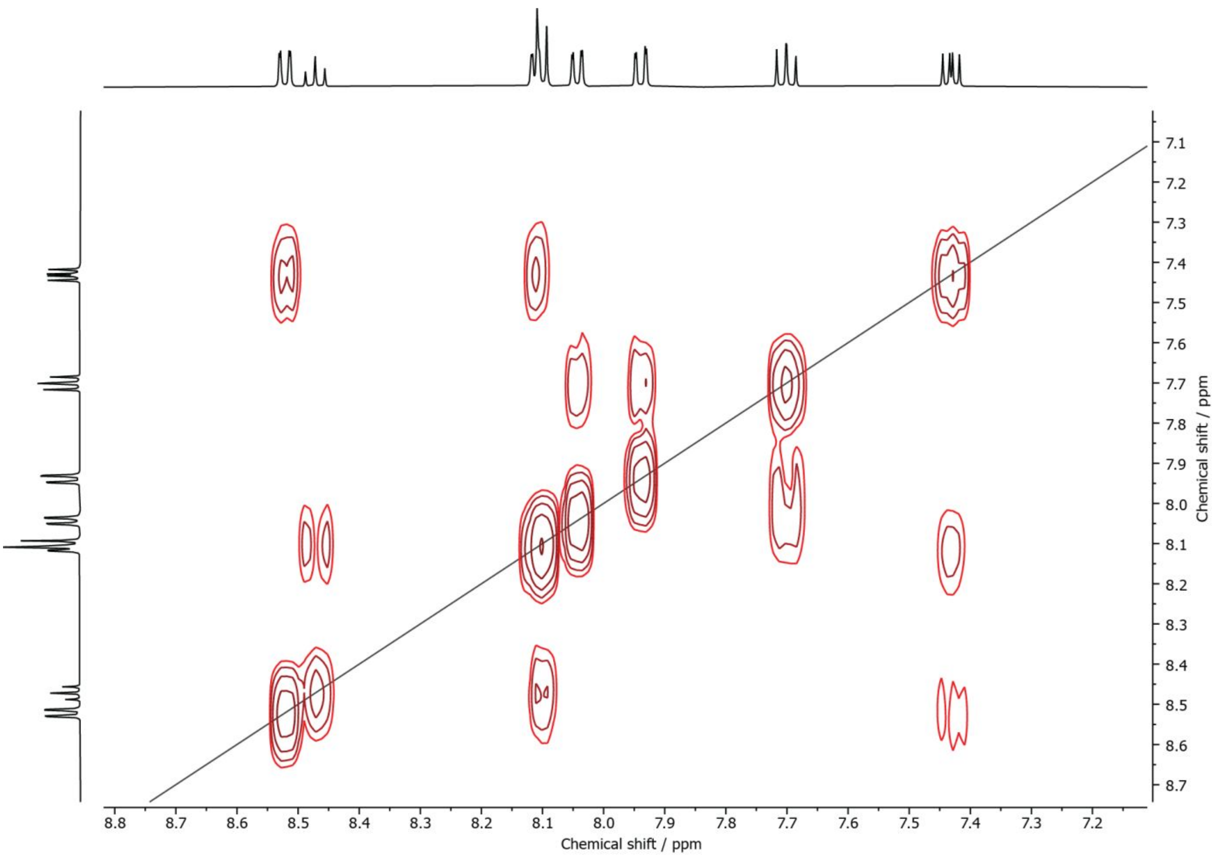

Figure S14. ^1H-^1H COSY NMR spectrum of $[Co(dqp)_2](PF_6)_3$ in CD_3CN.

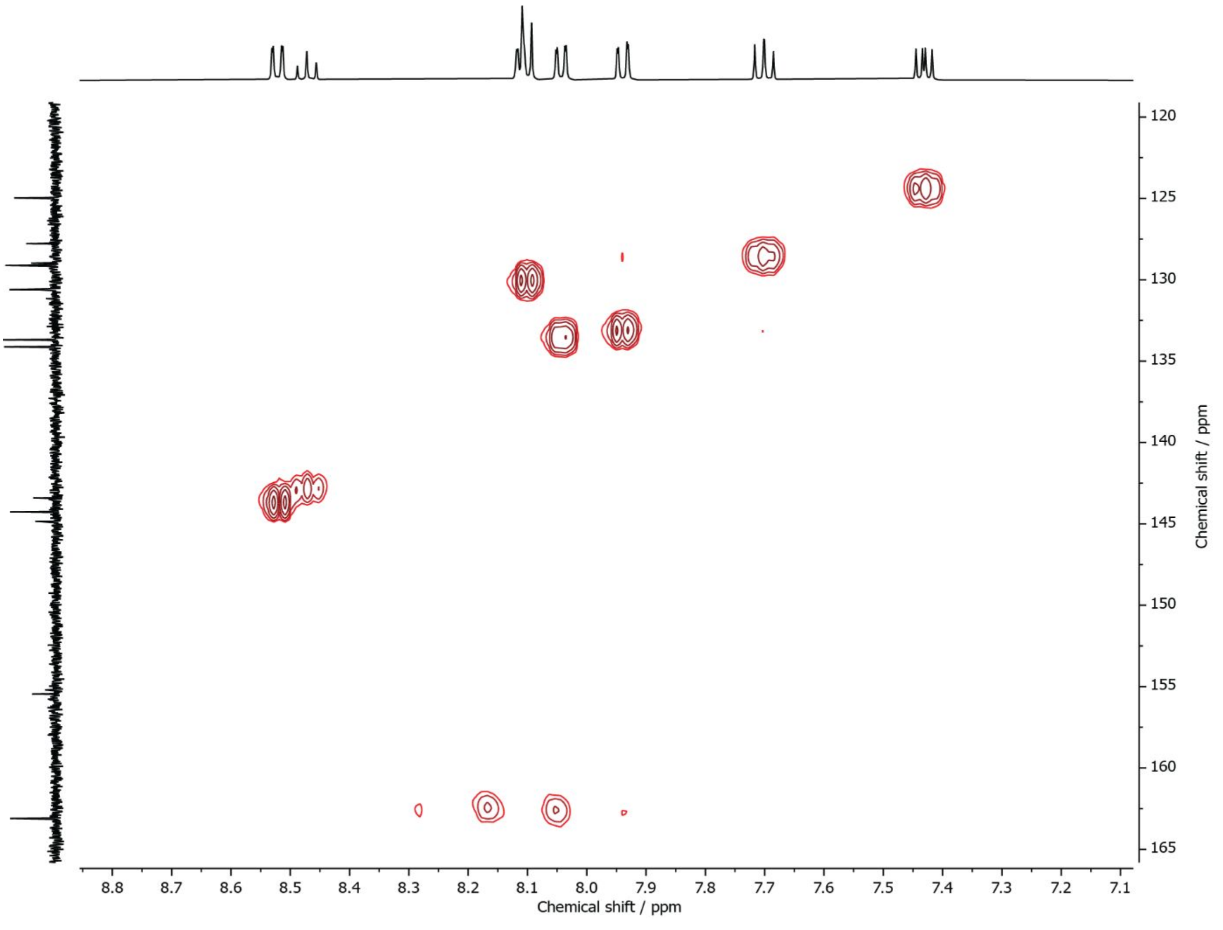

Figure S15. ^1H-^{13}C HMQC NMR spectrum of $[Co(dqp)_2](PF_6)_3$ in CD_3CN.

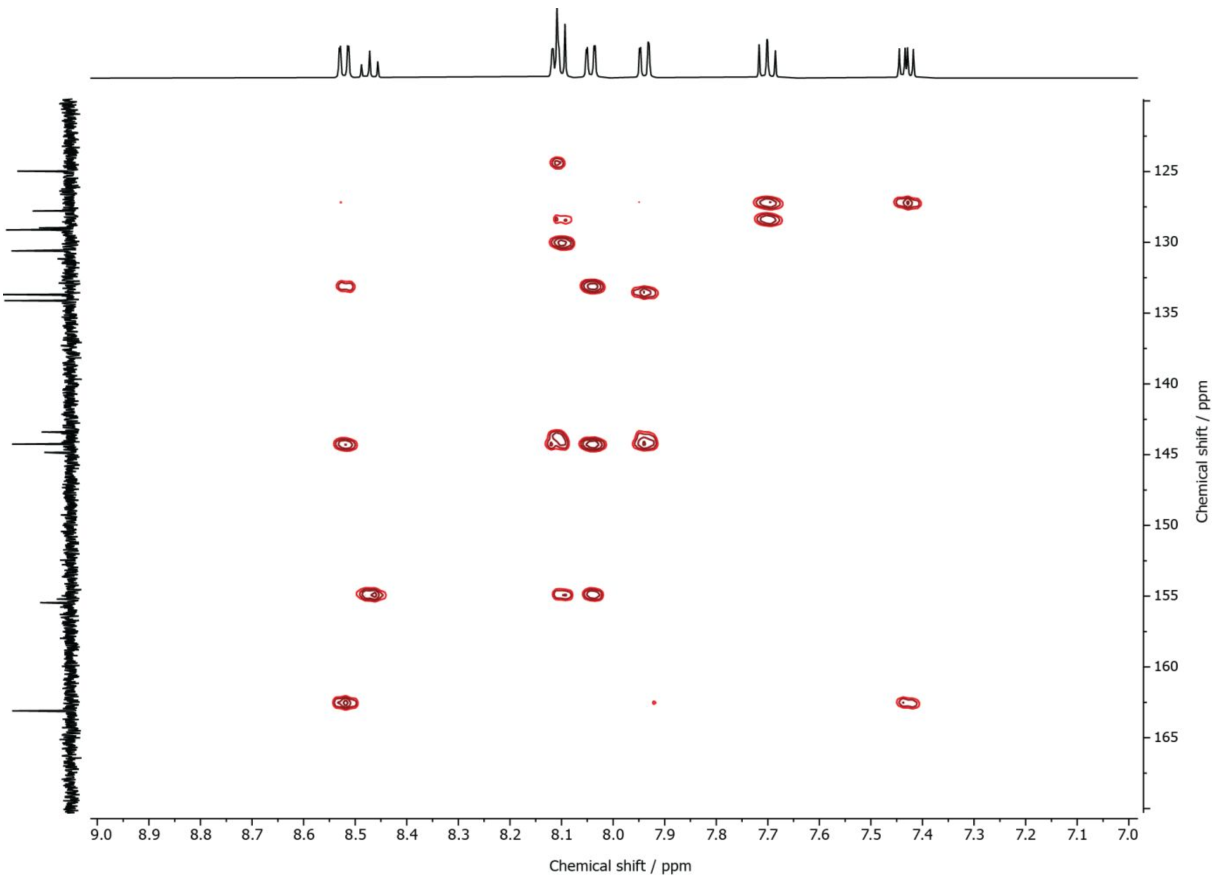

Figure S16. ^{1}H-^{13}C HMBC NMR spectrum of $[Co(dqp)_2](PF_6)_3$ in CD_3CN.

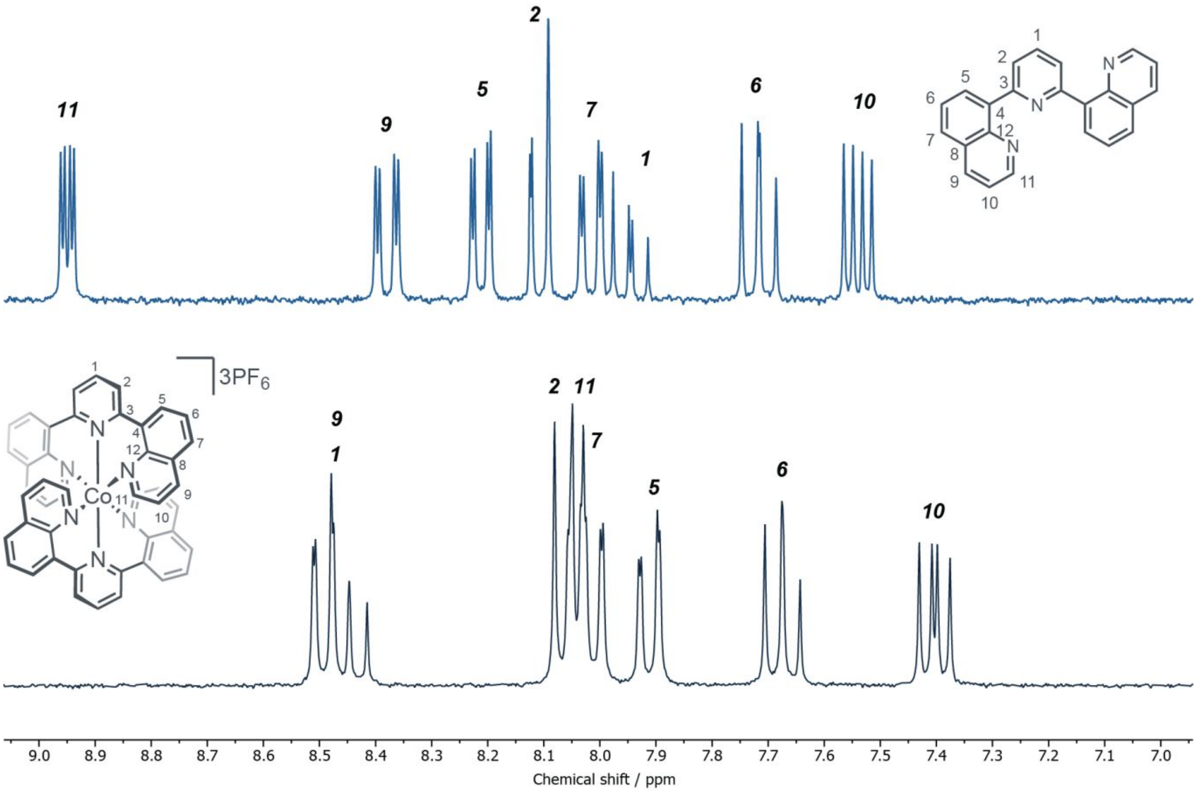

Figure S17. ^{1}H NMR (250 MHz, 298 K) spectrum of dqp in CD_3CN (top), $[Co(dqp)_2](PF_6)_3$ in CD_3CN (bottom).

Mass spectra

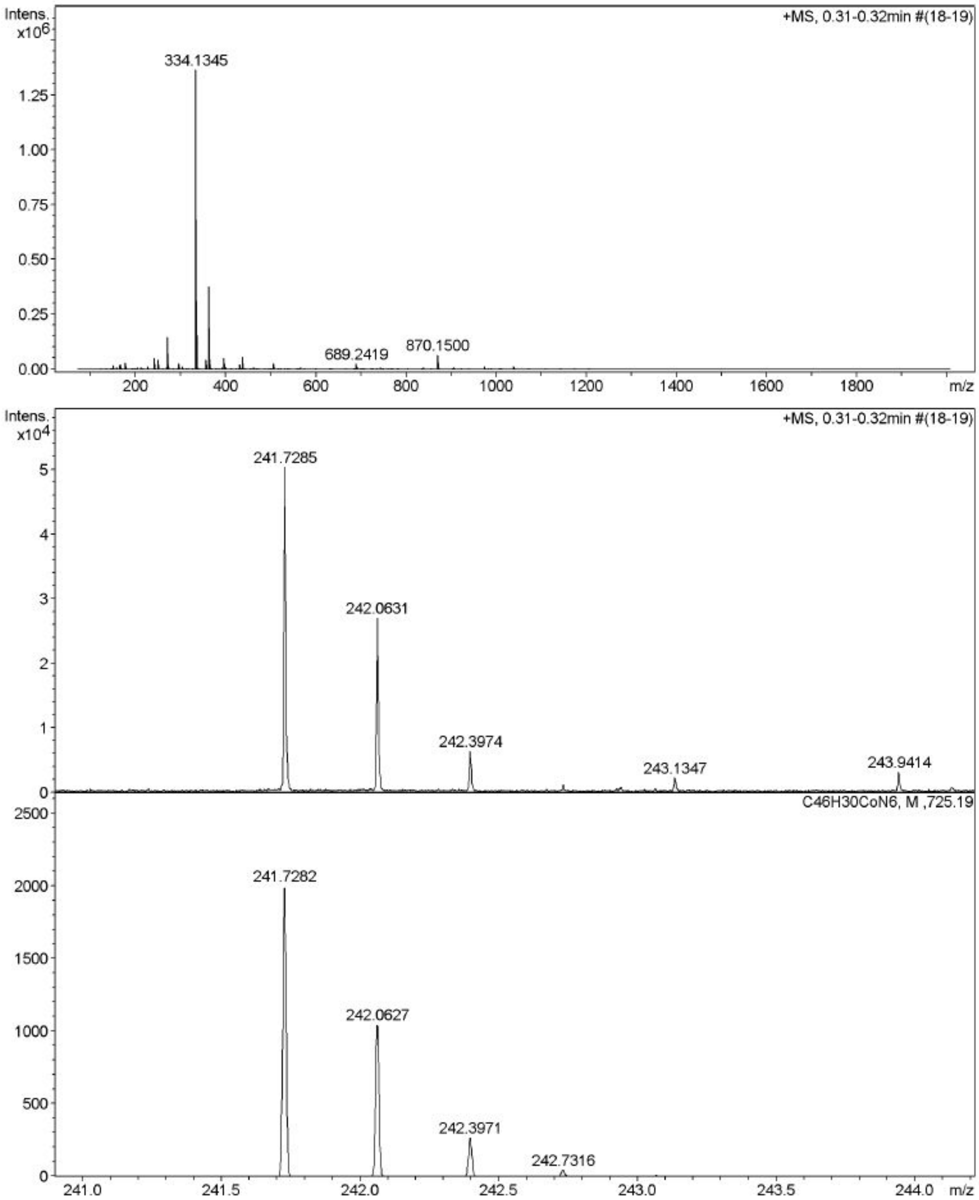

Figure S18. Experimental and calculated ESI(+)-HRMS spectra of $[Co(dqp)_2](PF_6)_3$.

Quantum chemistry

Computational details

All quantum chemical calculations to assess structural and electronic properties of the four Co^{III} complexes $[Co(phtpy)_2]^{3+}$, $[Co(dqp)_2]^{3+}$ were carried out using the Gaussian 16 program. Density functional theory (DFT) was employed to obtain fully relaxed ground state geometries of all four complexes within the singlet ground state (S_0) as well as within the lowest triplet state (T_1). To this aim, the B3LYP XC functional was applied in combination with the all-electron def2-SVP basis set.[15–18] Furthermore, dispersion correction was included in all simulations using the GD3 model with Becke-Johnson damping.[19] Implicit solvent effects (acetonitrile, $\varepsilon = 35.688$) were taken into account by the polarizable continuum model (PCM) using equilibrium procedure of the SMD solvation model.[20] All obtained geometries are minima of the $3N$-6-dimensional potential energy surface as verified by means of the performed vibrational analysis. All optimized structures are available via the free online repository Zenodo.[21]

Subsequently, time-dependent DFT (TD-DFT) calculations were carried out to evaluate the singlet and triplet excited states properties such as electronic characters, energies and oscillator strengths. To this aim, the same computation setup was employed as in the initial ground state calculations. The excited state properties within the Franck-Condon point (S_0 equilibrium structure) were evaluated by means of the non-equilibrium procedure of solvation to estimate the initial vertical absorption energies. The 100 lowest energy singlet-singlet excitations were obtained to model the electronic absorption spectra of $[Co(phtpy)_2]^{3+}$, $[Co(dqp)_2]^{3+}$ as well as the 100 lowest energy (dipole-forbidden) singlet-triplet transitions to evaluate prominent triplet states involved in the subsequent intersystem crossing (ISC) and excited state relaxation pathways.

Furthermore, the transition absorption (TA) spectra of the three complexes were simulated, where the excited-state absorption (ESA) was modelled by means of the lowest 150 spin and dipole-allowed triplet-triplet transitions as obtained within the previously optimized T_1 equilibria. Assuming a 1:1 population of S_0 and T_1, the ESAs are given by the triplet-triplet excitation and the ground state bleach (GSB) by the singlet-singlet excitations within the singlet ground state equilibrium for $[Co(phtpy)_2]^{3+}$, $[Co(dqp)_2]^{3+}$ respectively. This approach allows to reliably model the TA signal upon excitation of the longest wavelength absorption band at long delay times.[22–24]

All optimized structures (S_0 and ^{3}MC and ^{3}MLCT) as obtained at the B3LYP level of theory as well as high-resolution images of charge density differences are available from the online repository Zenodo via Ref.[21].

Computational results

[Co(phtpy)$_2$]$^{3+}$

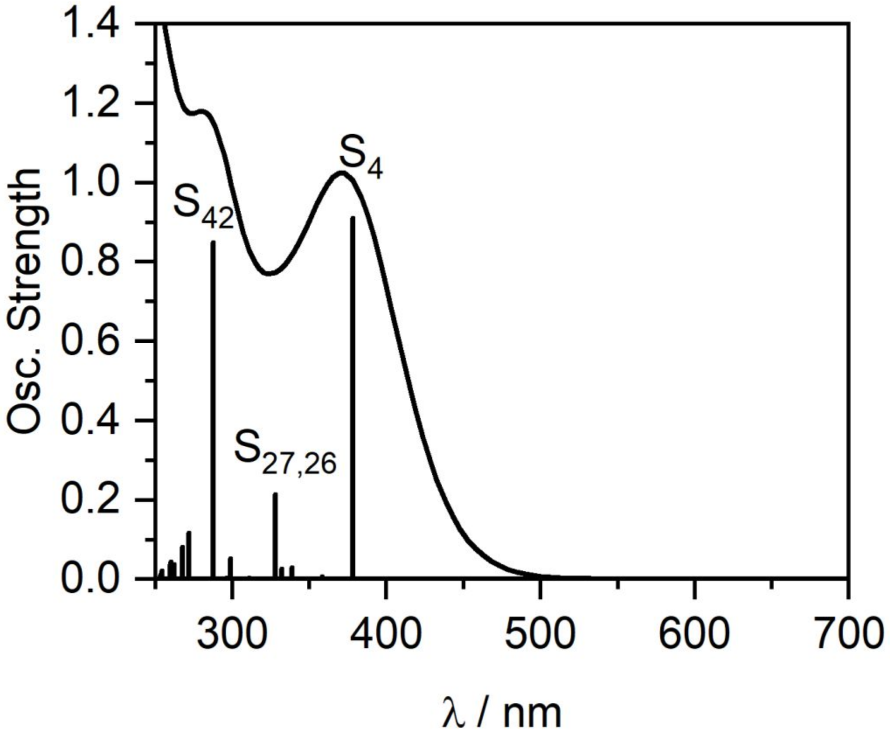

Figure S19. Simulated UV-Vis absorption spectrum of [Co(phtpy)$_2$]$^{3+}$; key electronic excitations contributing to the absorption are indicated. The transitions were broadened by Gaussian functions with a full width at half maximum of 0.2 eV.

Table S4. Simulated vertical excitation energies (E^e), wavelengths (λ), oscillator strengths (f), and singly-excited configurations of the singlet-singlet transitions as visualized by means of charge density difference (CDD) plots involved in the initial absorption of $[Co(phtpy)_2]^{3+}$ in the S_0 equilibrated geometry.

State	Transition Type	CDD	E^e / eV	λ / nm	f
S_1	MC, LMCT	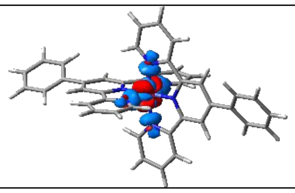	2.53	490	0.000
S_2	MC, LMCT	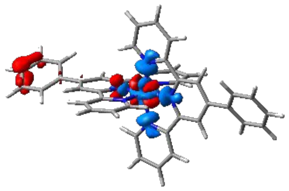	2.87	432	0.002
S_3	MC, LMCT	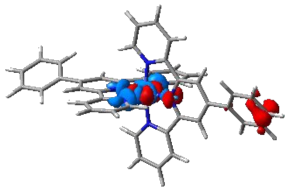	2.87	432	0.002
S_4	ILCT	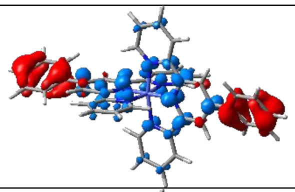	3.27	378	0.910
S_{26}	LC, LMCT	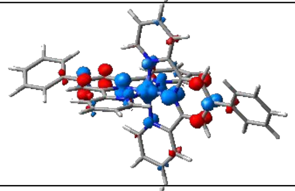	3.78	328	0.214
S_{42}	LC	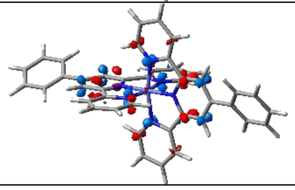	4.31	288	0.849

Table S5. Simulated vertical excitation energies (E^e), wavelengths (λ), oscillator strengths (f), and singly-excited configurations of the singlet-triplet transitions as visualized by means of charge density difference (CDD) plots involved in the initial absorption of $[Co(phtpy)_2]^{3+}$ in the S_0 equilibrated geometry.

State	Transition Type	CDD	E^e / eV	λ / nm	f	s^2
T_1	MC, LMCT	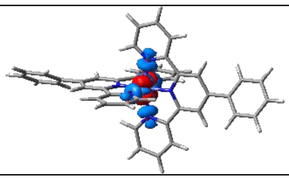	1.72	719	0.000	2.00
T_2	MC, LMCT	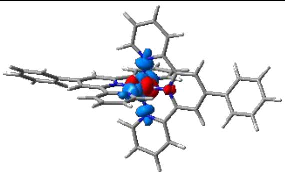	2.03	611	0.000	2.00
T_3	MC, LMCT	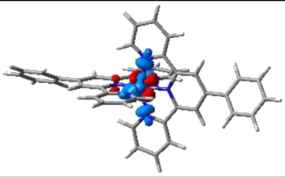	2.03	611	0.000	2.00
T_4	MC, LMCT	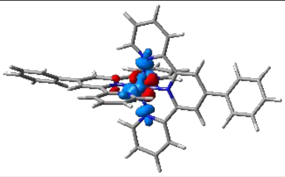	2.36	525	0.000	2.00
T_5	MC, LMCT	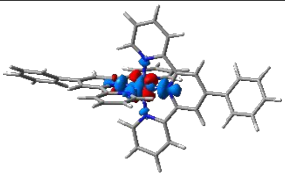	2.36	525	0.000	2.00
T_6	MC, LMCT	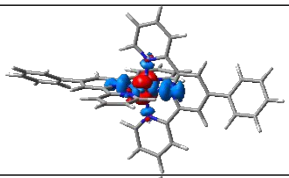	2.40	516	0.000	2.00
T_7	ILCT	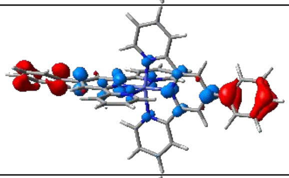	2.70	459	0.000	2.00
T_8	ILCT	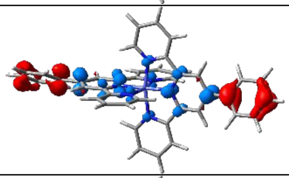	2.70	459	0.000	2.00
T_9	LC	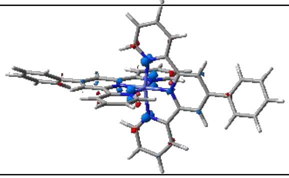	3.02	411	0.000	2.00

T_{10}	LC	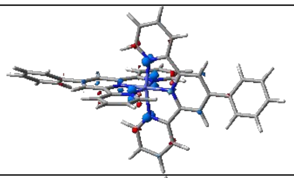	3.02	410	0.000	2.00
T_{11}	LC, ILCT	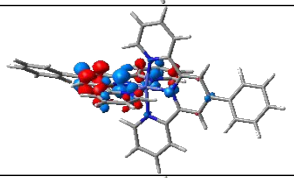	3.09	402	0.000	2.00
T_{12}	LC, ILCT	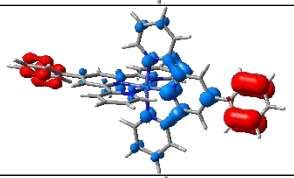	3.09	402	0.000	2.00
T_{13}	ILCT, LC	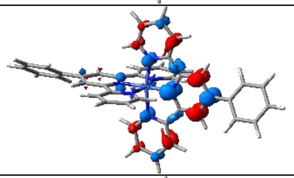	3.19	388	0.000	2.00
T_{14}	ILCT, LC	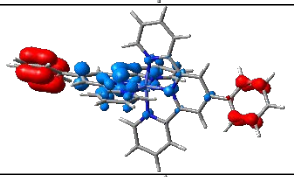	3.19	388	0.000	2.00
T_{15}	ILCT, LC	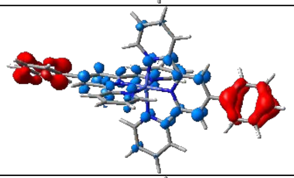	3.21	386	0.000	2.00
T_{16}	ILCT, LC	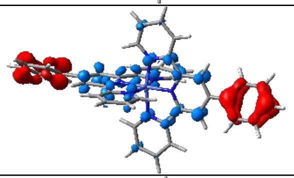	3.21	386	0.000	2.00
T_{17}	ILCT, LC	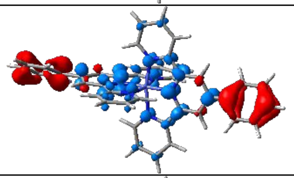	3.40	365	0.000	2.00
T_{18}	ILCT, LC	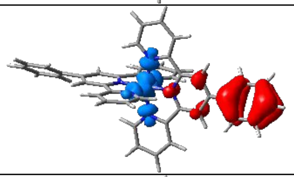	3.40	365	0.000	2.00
T_{19}	LMCT	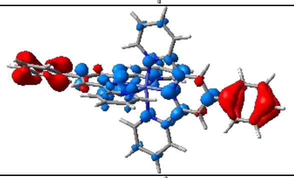	3.51	353	0.000	2.00
T_{20}	LMCT	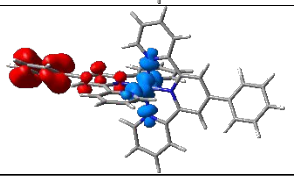	3.51	353	0.000	2.00

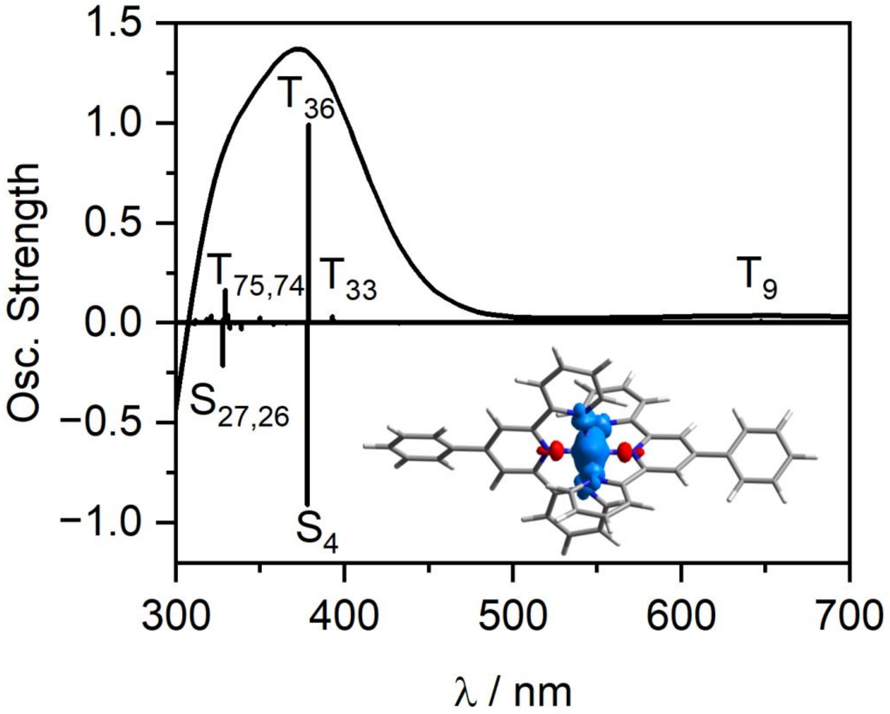

Figure S20. Simulated transient absorption spectrum of the $[Co(phtpy)_2]^+$; key electronic excitations contributing to the absorption are indicated. The transitions were broadened by Gaussian functions with a full width at half maximum of 0.2 eV. Spin density of the triplet state is displayed.

Table S6. Simulated vertical excitation energies (E^e), wavelengths (λ), oscillator strengths (f), and singly-excited configurations as visualized by means of charge density difference (CDD) plots of the spin and dipole-allowed triplet-triplet transitions involved in the excited-state absorption of $[Co(phtpy)_2]^{3+}$ within the T_1 geometry.

State	Transition Type	CDD	E^e / eV	λ / nm	f	s^2
T_9	LMCT	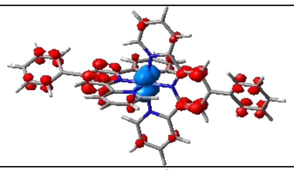	1.92	647	0.011	2.11
T_{33}	LMCT	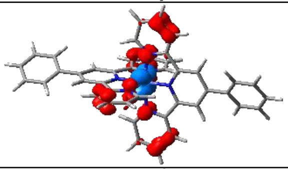	3.16	393	0.033	2.25
T_{36}	ILCT	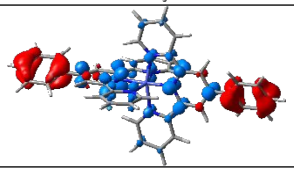	3.27	379	0.991	2.03
T_{74}	LC, ILCT, LMCT	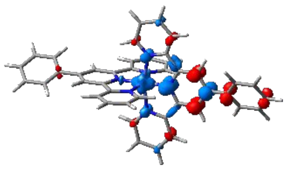	3.77	329	0.166	2.31
T_{75}	LC, ILCT, LMCT	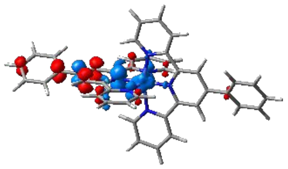	3.77	329	0.166	2.31
T_{77}	LMCT, ILCT	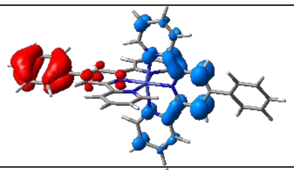	3.78	328	0.020	2.85
T_{78}	LMCT, ILCT	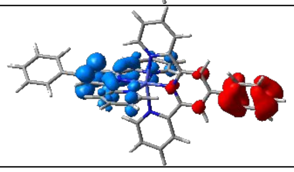	3.78	328	0.020	2.85

Table S7. Simulated vertical excitation energies (E^e), wavelengths (λ), oscillator strengths (f), and singly-excited configurations of the singlet-triplet transitions as visualized by means of charge density difference (CDD) plots involved in the initial absorption of $[Co(phtpy)_2]^{3+}$ within the T_1 structure.

State	Transition Type	CDD	E^e / eV	λ / nm	f	s^2
T_1	MC, LMCT	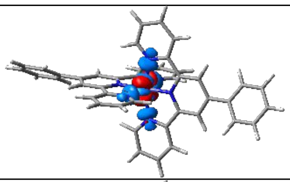	1.07	1161	0.000	2.00
T_2	MC, LMCT	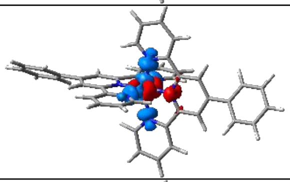	1.33	927	0.000	2.00
T_3	MC, LMCT	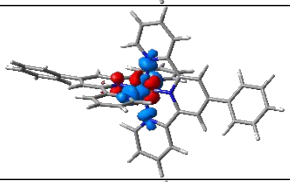	1.33	927	0.000	2.00
T_4	MC, LMCT	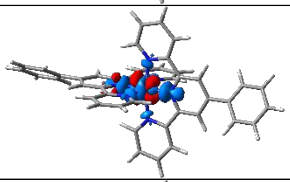	1.95	634	0.000	2.00
T_5	MC, LMCT	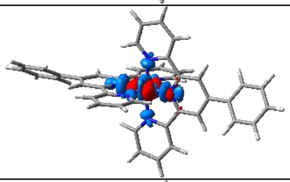	1.95	634	0.000	2.00
T_6	MC, LMCT	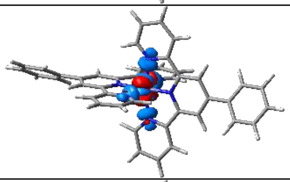	1.98	627	0.000	0.00
T_7	MC, LMCT	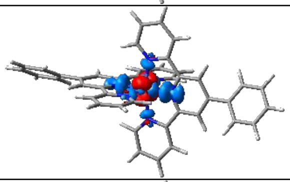	2.13	583	0.000	2.00
T_8	LMCT, MC	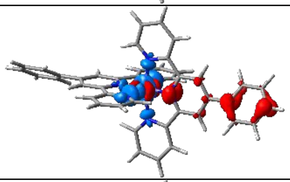	2.31	536	0.003	0.00
T_9	LMCT, MC	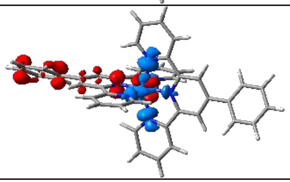	2.31	536	0.003	0.00

T_{10}	ILCT	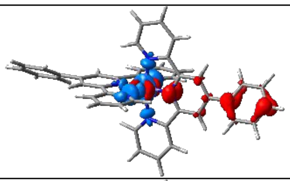	2.69	460	0.000	2.00
T_{11}	ILCT	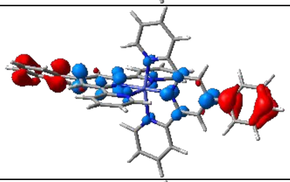	2.69	460	0.000	2.00
T_{12}	LMCT, MC	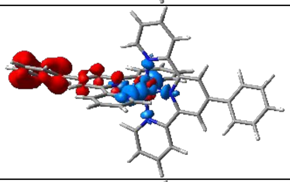	2.74	452	0.000	0.00
T_{13}	LMCT, MC	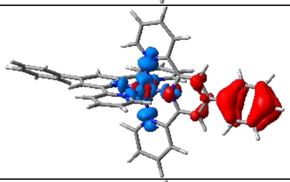	2.74	452	0.000	0.00
T_{14}	LMCT	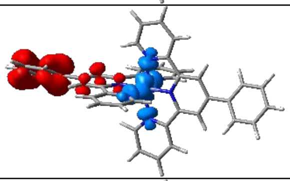	2.78	447	0.000	2.00
T_{15}	LMCT	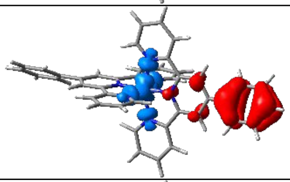	2.78	447	0.000	2.00
T_{16}	LMCT	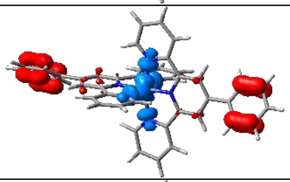	2.91	425	0.000	2.00
T_{17}	LMCT	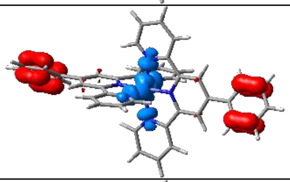	2.92	425	0.000	0.00
T_{18}	LMCT	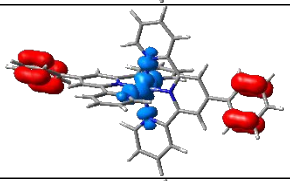	2.92	424	0.000	2.00
T_{19}	LMCT	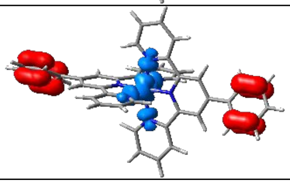	2.93	424	0.000	0.00

T_{20}	LMCT	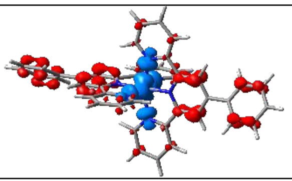	2.98	416	0.000	2.00

[Co(dqp)$_2$]$^{3+}$

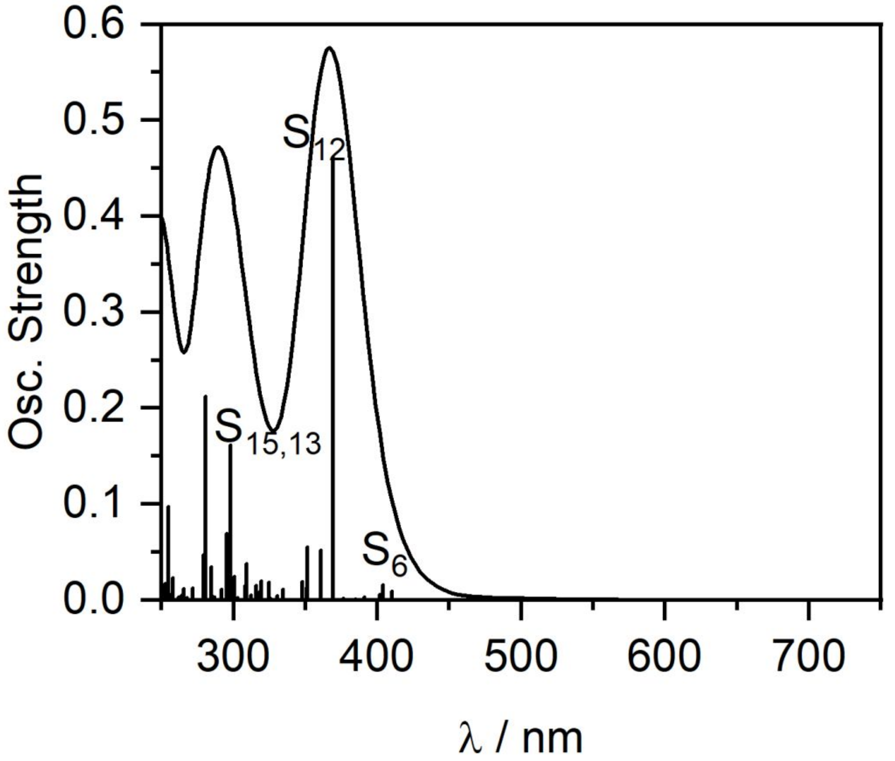

Figure S21. Simulated UV-Vis absorption spectrum of [Co(dqp)$_2$]$^{3+}$; key electronic excitations contributing to the absorption in the visible region are indicated. The transitions were broadened by Gaussian functions with a full width at half maximum of 0.2 eV.

Table S8. Simulated vertical excitation energies (E^e), wavelengths (λ), oscillator strengths (f), and singly-excited configurations of the singlet-singlet transitions as visualized by means of charge density difference (CDD) plots involved in the initial absorption of $[Co(dqp)_2]^{3+}$ in the S_0 equilibrated geometry.

State	Transition Type	CDD	E^e / eV	λ / nm	f
S_1	MC	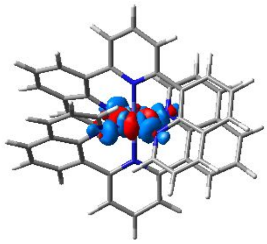	2.45	506	0.000
S_2	MC	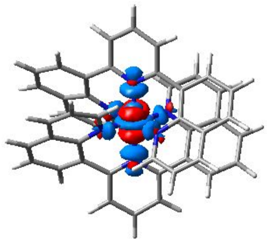	2.56	485	0.001
S_3	MC	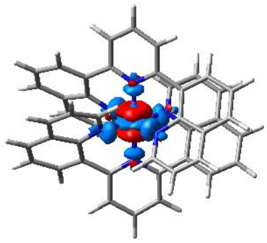	2.58	480	0.000
S_4	LMCT	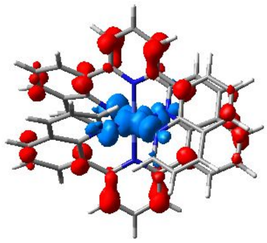	2.81	442	0.000
S_5	LMCT	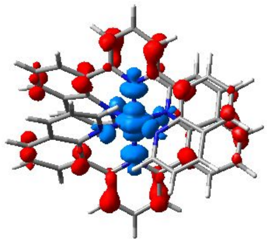	3.02	410	0.009
S_6	LC, LMCT	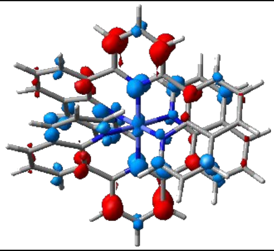	3.07	404	0.016

S_{12}	LC, MC	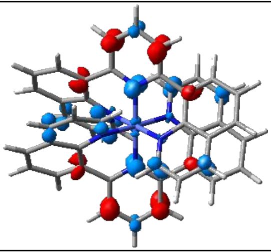	3.36	369	0.462
S_{13}	LMCT, LC	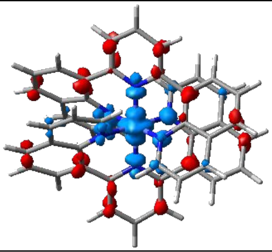	3.44	361	0.052
S_{15}	LC, MLCT	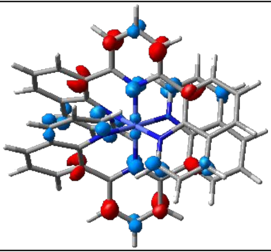	3.52	352	0.055

Table S9. Simulated vertical excitation energies (E^e), wavelengths (λ), oscillator strengths (f), and singly-excited configurations of the singlet-triplet transitions as visualized by means of charge density difference (CDD) plots involved in the initial absorption of $[Co(dqp)_2]^{3+}$ in the S_0 equilibrated geometry.

State	Transition Type	CDD	E^e / eV	λ / nm	f	s^2
T_1	MC, MLCT, LC	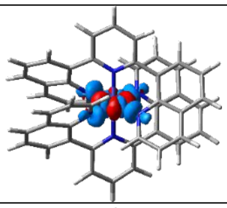	1.65	750	0.000	2.00
T_2	MC, LMCT, ILCT	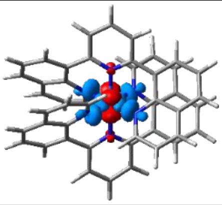	1.71	726	0.000	2.00
T_3	MC, LMCT, LC	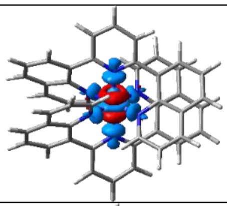	1.78	697	0.000	2.00
T_4	MC, LMCT, LC	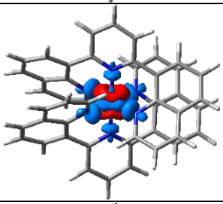	2.05	606	0.000	2.00
T_5	MC, LMCT, LC	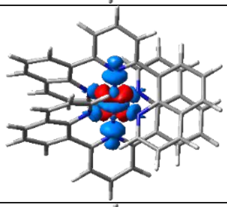	2.12	583	0.000	2.00
T_6	MC, LMCT, LC	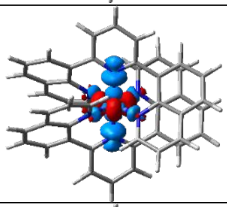	2.20	564	0.000	2.00
T_7	LMCT, LC	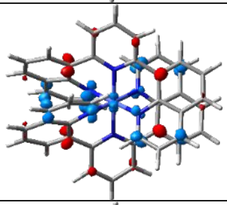	2.46	504	0.000	2.00
T_8	LC, LMCT	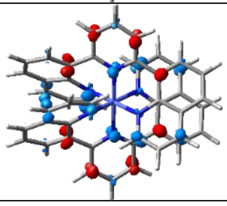	2.56	504	0.000	2.00

T_9	LC, LMCT	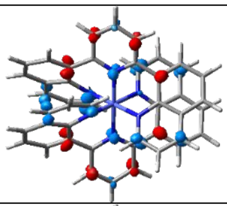	2.49	499	0.000	2.00
T_{10}	LMCT, LC	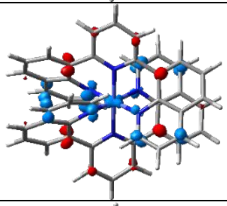	2.50	467	0.000	2.00
T_{11}	LMCT, LC	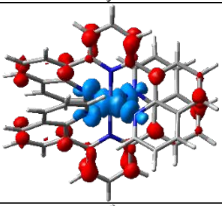	2.78	445	0.000	2.00
T_{12}	LMCT, LC	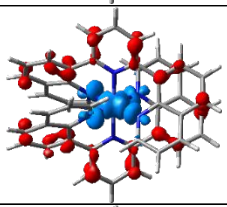	3.00	414	0.000	2.00
T_{13}	LMCT, LC	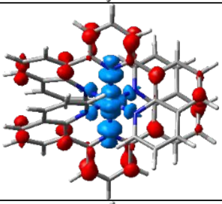	3.08	403	0.000	2.00
T_{14}	LMCT, LC	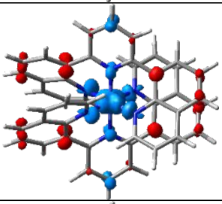	3.21	386	0.000	2.00
T_{15}	LC, LMCT	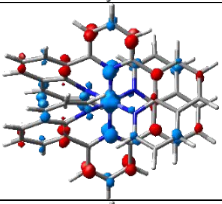	3.22	385	0.000	2.00
T_{16}	LC, LMCT	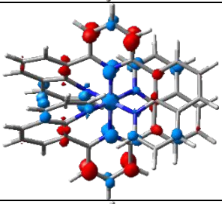	3.25	381	0.000	2.00
T_{17}	LMCT, LC	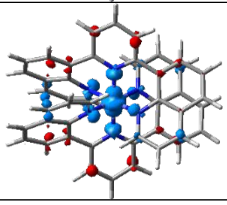	3.25	381	0.000	2.00

T_{18}	LC, LMCT	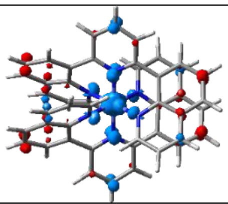	3.28	378	0.000	2.00
T_{19}	LMCT, LC	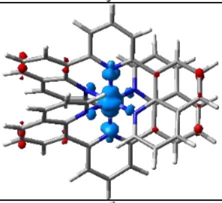	3.31	374	0.000	2.00
T_{20}	LC, LMCT	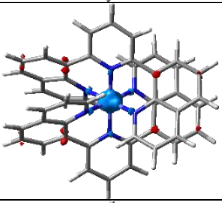	3.32	373	0.000	2.00
T_{21}	LC, LMCT	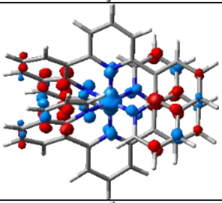	3.34	371	0.000	2.00
T_{22}	LMCT, LC	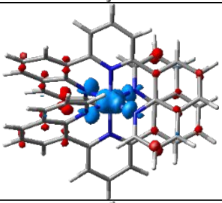	3.34	371	0.000	2.00
T_{23}	LC, LMCT	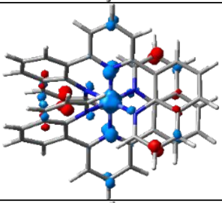	3.39	365	0.000	2.00
T_{24}	LMCT, LC	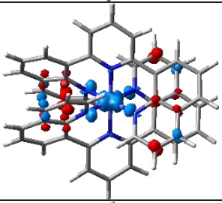	3.40	365	0.000	2.00
T_{25}	LMCT, LC	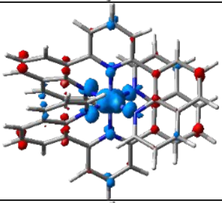	3.45	360	0.000	2.00
T_{26}	LMCT, MC, LC	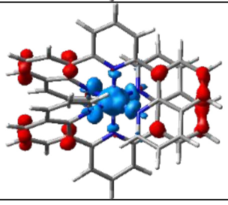	3.52	353	0.000	2.00

T_{27}	LMCT, LC	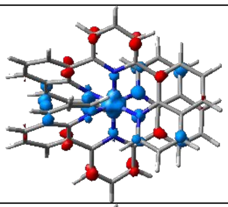	3.54	350	0.000	2.00
T_{28}	LMCT, LC	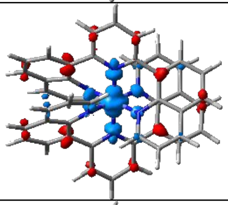	3.61	343	0.000	2.00
T_{29}	LMCT, LC	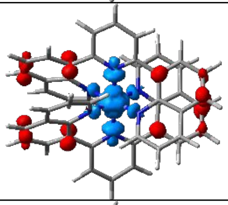	3.62	343	0.000	2.00
T_{30}	LC	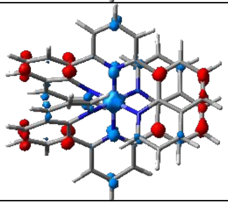	3.63	342	0.000	2.00

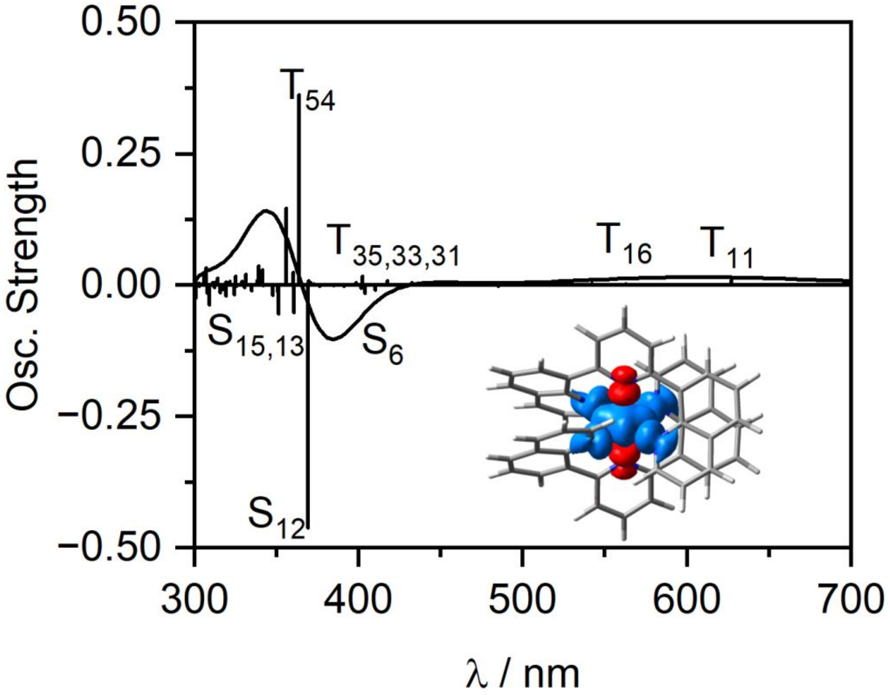

Figure S22. Simulated transient absorption spectrum of the $[Co(dqp)_2]^{3+}$; key electronic excitations contributing to the absorption are indicated. The transitions were broadened by Gaussian functions with a full width at half maximum of 0.2 eV. Spin density of the triplet state is displayed.

Table S10. Simulated vertical excitation energies (E^e), wavelengths (λ), oscillator strengths (f), and singly-excited configurations as visualized by means of charge density difference (CDD) plots of the spin and dipole-allowed triplet-triplet transitions involved in the excited-state absorption of $[Co(dqp)_2]^{3+}$ within the T_1 geometry.

State	Transition Type	CDD	E^e / eV	λ / nm	f	s^2
T_{11}	LMCT	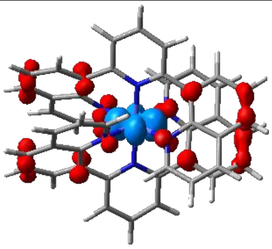	1.98	627	0.010	2.12
T_{16}	LMCT, MC	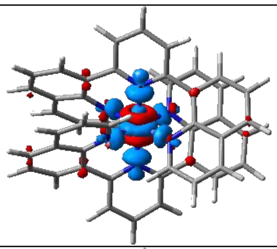	2.20	563	0.003	2.41
T_{31}	MC, LMCT	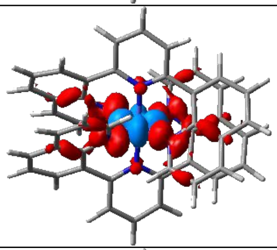	2.87	432	0.005	2.08
T_{33}	LMCT, MC	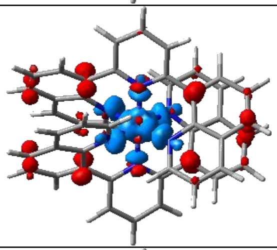	2.97	418	0.009	2.29
T_{35}	LC	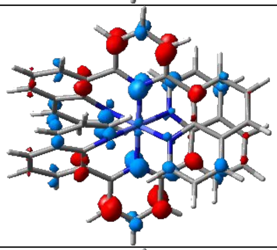	3.08	402	0.018	2.05
T_{54}	LC	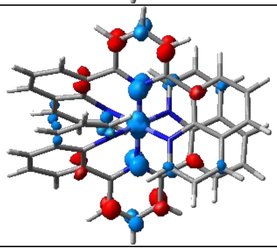	3.41	364	0.363	2.17

Table S11. Simulated vertical excitation energies (E^e), wavelengths (λ), oscillator strengths (f), and singly-excited configurations of the singlet-triplet transitions as visualized by means of charge density difference (CDD) plots involved in the initial absorption of $[Co(dqp)_2]^{3+}$ within the T_1 structure.

State	Transition Type	CDD	E^e / eV	λ / nm	f	s^2
T_1	MC, LMCT	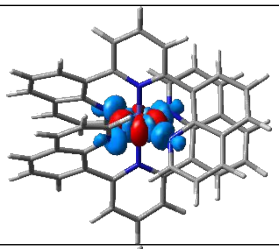	1.00	1283	0.000	2.00
T_2	MC, LMCT	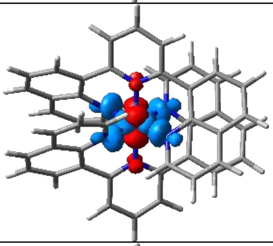	1.02	1210	0.000	2.00
T_3	MC, LMCT	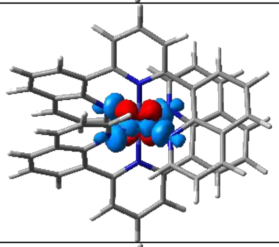	1.22	1009	0.000	2.00
T_4	MC, LMCT	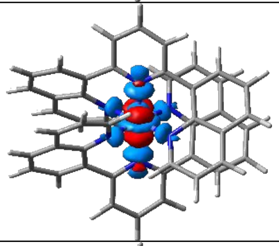	1.71	723	0.000	2.00
T_5	MC, LMCT	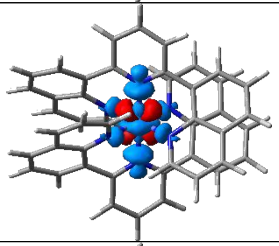	1.92	644	0.000	2.00
T_6	MC, LMCT	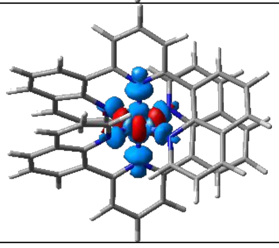	2.08	596	0.000	2.00

T_7	LMCT	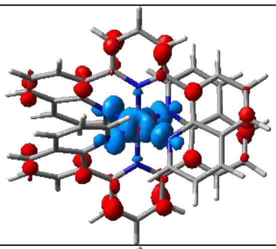	2.11	588	0.000	2.00
T_8	LMCT	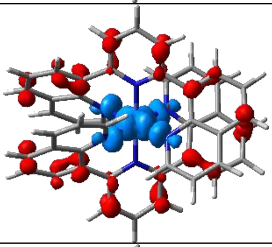	2.34	530	0.000	2.00
T_9	LC	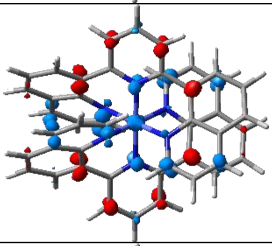	2.47	502	0.000	2.00
T_{10}	LMCT, LC	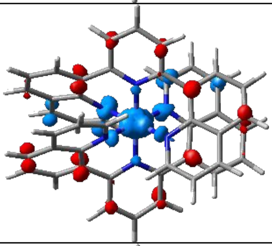	2.49	498	0.000	2.00
T_{11}	LMCT, LC	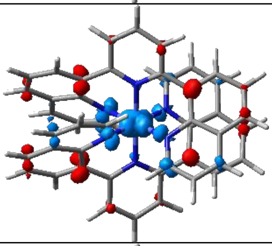	2.49	498	0.000	2.00
T_{12}	LC, LMCT	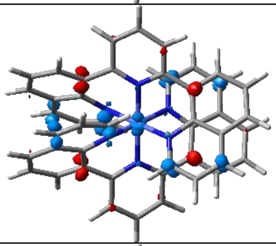	2.55	487	0.000	2.00
T_{13}	LMCT, LC	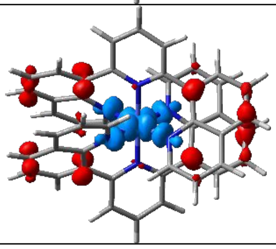	2.63	472	0.000	2.00

T_{14}	LMCT	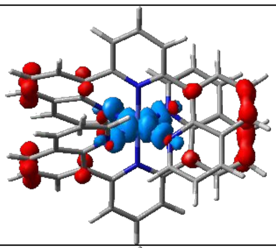	2.82	439	0.000	2.00
T_{15}	LMCT, LC	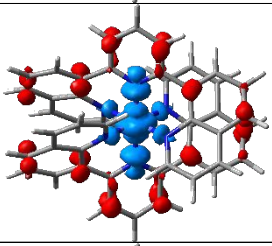	2.83	438	0.000	2.00
T_{16}	LMCT	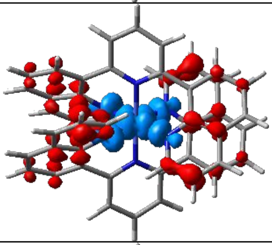	2.97	418	0.000	2.00
T_{17}	LMCT	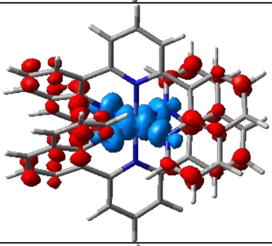	2.99	414	0.000	2.00
T_{18}	LMCT	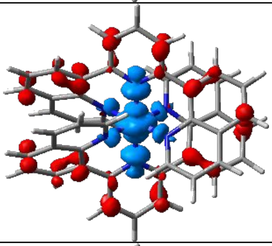	3.04	408	0.000	2.00
T_{19}	LMCT	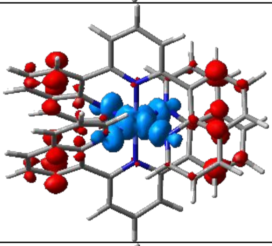	3.10	400	0.000	2.00
T_{20}	LMCT	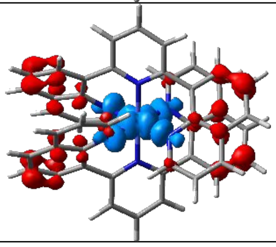	3.11	399	0.000	2.00

T_{21}	LMCT, LC	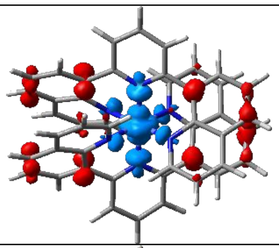	3.21	387	0.000	2.00
T_{22}	LMCT, LC	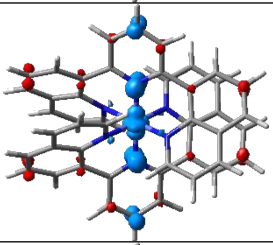	3.28	378	0.000	2.00
T_{23}	LC, LMCT	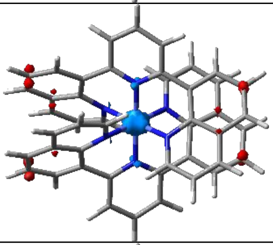	3.30	376	0.000	2.00
T_{24}	LMCT, LC	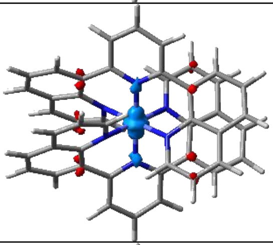	3.32	373	0.000	2.00
T_{25}	LC	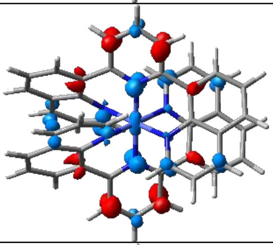	3.33	372	0.000	2.00
T_{26}	LC	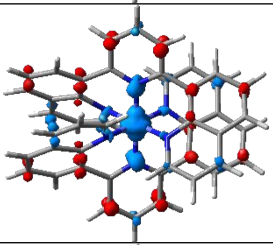	3.34	372	0.000	2.00
T_{27}	LMCT, LC	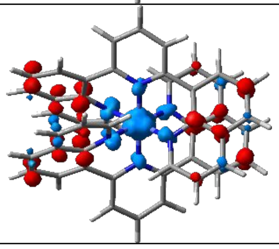	3.40	364	0.000	2.00

T_{28}	LMCT, LC	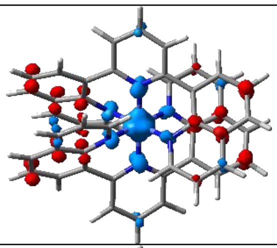	3.42	362	0.000	2.00
T_{29}	LMCT, MC	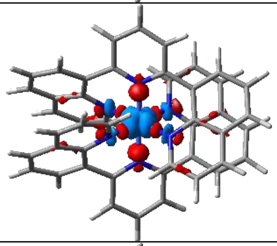	3.46	359	0.000	2.00
T_{30}	LC, LMCT	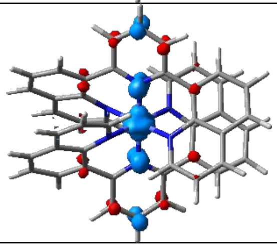	3.46	359	0.000	2.00

Table S12. Simulated Co-N bond lengths and ligand bite angles for $[Co(phtpy)_2]^{3+}$ and $[Co(dqp)_2]^{3+}$ in fully relaxed S_0 and T_1 geometries.

	$[Co(phtpy)_2]^{3+}$		$[Co(dqp)_2]^{3+}$	
	S_0	T_1	S_0	T_1
Co-N_1 (Å)	1.9585	2.0576	1.9784	2.0869
Co-N_2 (Å)	1.8698	1.8880	1.9611	1.9513
Co-N_3 (Å)	1.9585	2.0576	1.9783	2.0867
Co-N_4 (Å)	1.9585	2.0576	1.9783	2.0857
Co-N_5 (Å)	1.8698	1.8880	1.9612	1.9515
Co-N_6 (Å)	1.9585	2.0576	1.9783	2.0859
Bite angle (Å)	165.0	162.4	178.7	179.9
		162.4		180.0

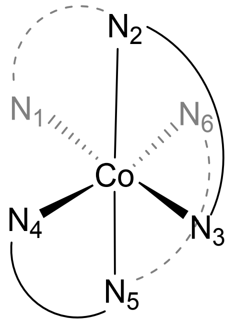

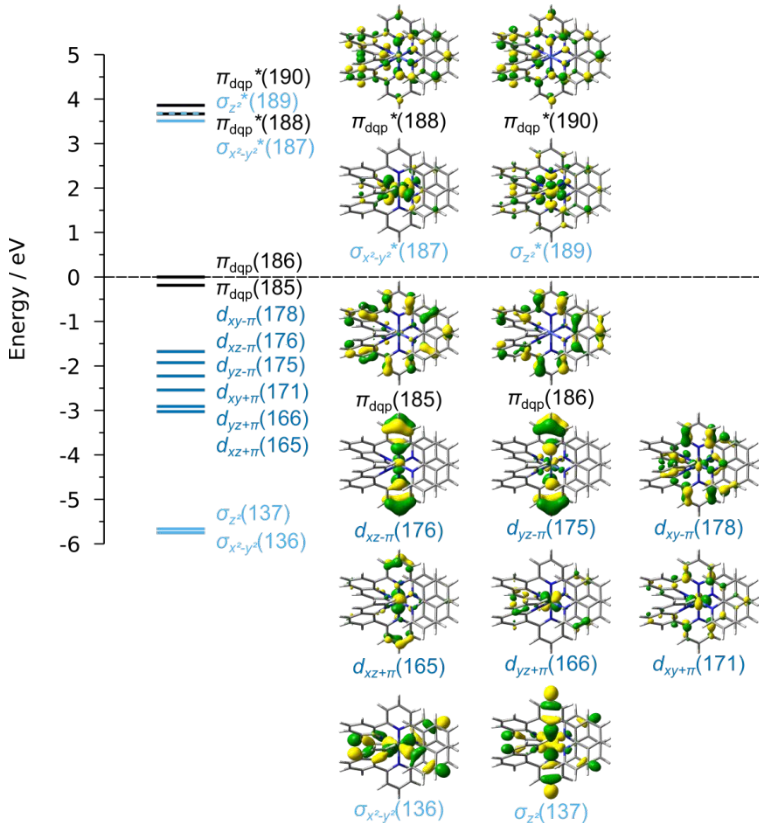

Figure S23. Energy diagram of the frontier molecular orbitals of $[Co(dqp)_2]^{3+}$. High resolution images as well as cube-files are available via Zenodo.[21]

Table S13. Frontier molecular orbitals and respective energies of $[Co(dqp)_2]^{3+}$.; all energies are shown relative to the highest-occupied molecular orbital π_{dqp}(186). High resolution images as well as cube-files are available via Zenodo.[21]

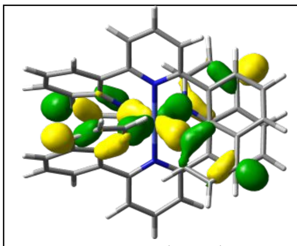 $\sigma_{x^2-y^2}(136)$, -5.75 eV	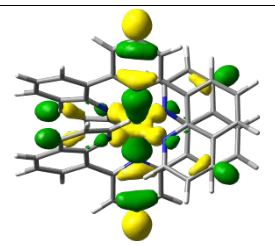 $\sigma_{z^2}(137)$, -5.66 eV	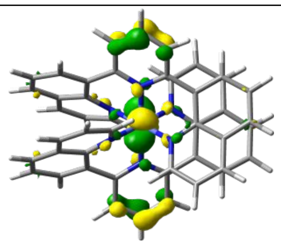 $d_{xz+\pi}(165)$, -3.03 eV	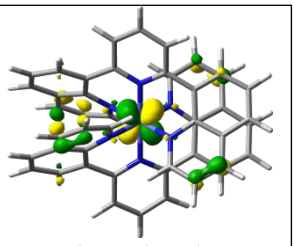 $d_{yz+\pi}(166)$, -2.91 eV
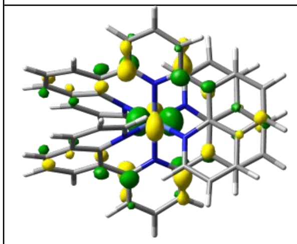 $d_{xy+\pi}(171)$, -2.54 eV	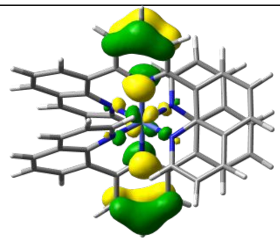 $d_{yz-\pi}(175)$, -2.23 eV	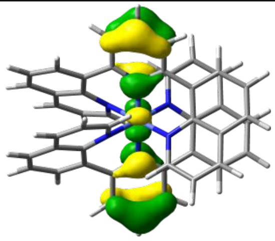 $d_{xz-\pi}(176)$, -1.93 eV	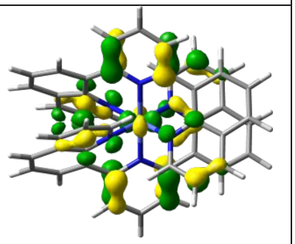 $d_{xy-\pi}(178)$, -1.68 eV
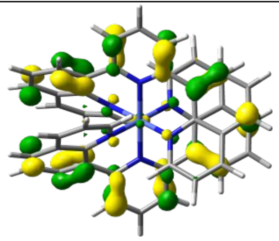 $\pi_{\mathrm{dqp}}(185)$, -0.19 eV	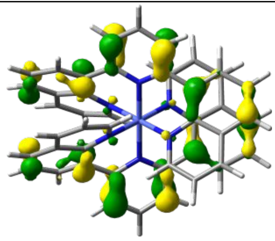 $\pi_{\mathrm{dqp}}(186)$, 0.00 eV	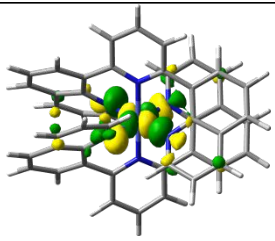 $\sigma^*_{x^2-y^2}(187)$, 3.51 eV	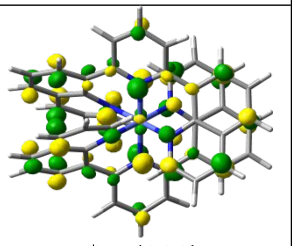 $\pi^*_{\mathrm{dqp}}(188)$, 3.67 eV
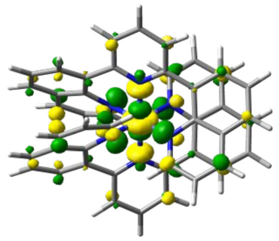 $\sigma^*_{z^2}(189)$, 3.68 eV	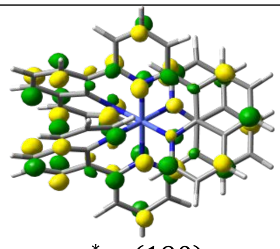 $\pi^*_{\mathrm{dqp}}(190)$, 3.86 eV		

References

(1) Constable, E. C.; Harris, K.; Housecroft, C. E.; Neuburger, M.; Zampese, J. A. Turning $\{M(tpy)_2\}^{n+}$ embraces and CH···π interactions on and off in homoleptic cobalt(II) and cobalt(III) bis(2,2′:6′,2″-terpyridine) complexes. *CrystEngComm* **2010**, *12*, 2949.

(2) Jäger, M.; Eriksson, L.; Bergquist, J.; Johansson, O. Synthesis and Characterization of 2,6-Di(Quinolin-8-Yl)Pyridines. New Ligands for Bistridentate Ru^{II} Complexes with Microsecond Luminescent Lifetimes. *J. Org. Chem.* **2007**, *72*, 10227–10230.

(3) Gottlieb, H. E.; Kotlyar, V.; Nudelman, A. NMR Chemical Shifts of Common Laboratory Solvents as Trace Impurities. *J. Org. Chem.* **1997**, *62,* 7512–7515.

(4) Pal, A. K.; Li, C.; Hanan, G. S.; Zysman-Colman, E. Blue-Emissive Cobalt(III) Complexes and Their Use in the Photocatalytic Trifluoromethylation of Polycyclic Aromatic Hydrocarbons. *Angew. Chem. Int. Ed.* **2018**, *57*, 8027–8031.

(5) Garakyaraghi, S.; Danilov, E. O.; McCusker, C. E.; Castellano, F. N. Transient Absorption Dynamics of Sterically Congested Cu(I) MLCT Excited States. *J. Phys. Chem. A* **2015**, *119*, 3181–3193.

(6) Ortiz, R. J.; Mondal, R.; McCusker, J. K.; Herbert, D. E. Leveraging Intramolecular π-Stacking to Access an Exceptionally Long-Lived ^{3}MC Excited State in an Fe(II) Carbene Complex. *J. Am. Chem. Soc.* **2025**, *147*, 1694–1708.

(7) Fielding, L. Determination of Association Constants (Ka) from Solution NMR Data. *Tetrahedron* **2000**, *56*, 6151–6170.

(8) Clarke, R. H.; Hochstrasser, R. M. Location and Assignment of the Lowest Triplet State of Perylene. *J. Mol. Spectrosc.* **1969**, *32*, 309–319.

(9) Ni, W.; Sun, L.; Gurzadyan, G. G. Ultrafast Spectroscopy Reveals Singlet Fission, Ionization and Excimer Formation in Perylene Film. *Sci. Rep.* **2021**, *11*, 5220.

(10) Wegeberg, C.; Sinha, N.; Häussinger, D.; Prescimone, A.; Wenger, O. S. Photoredox-active Cr(0) luminophores featuring photophysical properties competitive with Ru(II) and Os(II) complexes. *Nat. Chem.* **2023**, *15*, 1730–1736.

(11) Turner, J. M.; Karl, M. W.; Kauffman, J. F. Spectroscopic Signatures of Protonated Perylene in Concentrated Sulfuric Acid. *J. Photochem. Photobiol. A Chem.* **2004**, *163*, 433–438.

(12) Dolomanov, O. V.; Bourhis, L. J.; Gildea, R. J.; Howard, J. A. K.; Puschmann, H. *OLEX2* : A Complete Structure Solution, Refinement and Analysis Program. *J. Appl. Crystallogr.* **2009**, *42*, 339–341.

(13) Sheldrick, G. M. *SHELXT* – Integrated Space-Group and Crystal-Structure Determination. *Acta. Crystallogr. A Found. Adv.* **2015**, *71*, 3–8.

(14) Sheldrick, G. M. Crystal Structure Refinement with *SHELXL*. *Acta Crystallogr. C Struct. Chem.* **2015**, *71*, 3–8.

(15) Becke, A. D. Density-Functional Thermochemistry. III. The Role of Exact Exchange. *J. Chem. Phys.* **1993**, *98*, 5648–5652.

(16) Lee, C.; Yang, W.; Parr, R. G. Development of the Colle-Salvetti Correlation-Energy Formula into a Functional of the Electron Density. *Phys. Rev. B* **1988**, *37*, 785–789.

(17) Weigend, F. Accurate Coulomb-Fitting Basis Sets for H to Rn. *Phys. Chem. Chem. Phys.* **2006**, *8*, 1057-1065.

(18) Weigend, F.; Ahlrichs, R. Balanced Basis Sets of Split Valence, Triple Zeta Valence and Quadruple Zeta Valence Quality for H to Rn: Design and Assessment of Accuracy. *Phys. Chem. Chem. Phys.* **2005**, *7*, 3297-3305.

(19) Grimme, S.; Ehrlich, S.; Goerigk, L. Effect of the Damping Function in Dispersion Corrected Density Functional Theory. *J. Comput. Chem.* **2011**, *32*, 1456–1465.

(20) Mennucci, B.; Cappelli, C.; Guido, C. A.; Cammi, R.; Tomasi, J. Structures and Properties of Electronically Excited Chromophores in Solution from the Polarizable Continuum Model Coupled to the Time-Dependent Density Functional Theory. *J. Phys. Chem. A* **2009**, *113*, 3009–3020.

(21) Kupfer, S.; Maisuradze, T. Quantum Chemical Data: Structural Control of Metal-Centered Excited States in Cobalt(III) Complexes via Bite Angle and π-π Interactions; Zenodo: **2025**, DOI: 10.5281/zenodo.15365044.

(22) Witas, K.; Nair, S. S.; Maisuradze, T.; Zedler, L.; Schmidt, H.; Garcia-Porta, P.; Rein, A. S. J.; Bolter, T.; Rau, S.; Kupfer, S.; Dietzek-Ivanšić, B.; Sorsche, D. U. Beyond the First Coordination Sphere─Manipulating the Excited-State Landscape in Iron(II) Chromophores with Protons. *J. Am. Chem. Soc.* **2024**, *146*, 19710–19719.

(23) Wegeberg, C.; Häussinger, D.; Kupfer, S.; Wenger, O. S. Controlling the Photophysical Properties of a Series of Isostructural d^6 Complexes Based on Cr^0, Mn^I, and Fe^{II}. *J. Am. Chem. Soc.* **2024**, *146*, 4605–4619.

(24) Sinha, N.; Wellauer, J.; Maisuradze, T.; Prescimone, A.; Kupfer, S.; Wenger, O. S. Reversible Photoinduced Ligand Substitution in a Luminescent Chromium(0) Complex. *J. Am. Chem. Soc.* **2024**, *146*, 10418–10431.
